# Nuclear protein 1 is a cell death regulator in primary human airway epithelial cells and reduced in idiopathic pulmonary fibrosis

**DOI:** 10.1038/s41598-026-51510-1

**Published:** 2026-05-11

**Authors:** Marie Zöller, Michal Mastalerz, Elisabeth Dick, Juliane Merl-Pham, Elisabeth Hennen, Sai Rama Sridatta Prakki, Ashesh Chakraborty, Misako Nakayama, Markus Klotz, Hannah Marchi, Ronan LeGleut, Laurens J. De Sadeleer, Wim A. Wuyts, Bart M. Vanaudenaerde, Aicha Jeridi, Antje Prasse, Benedikt Jäger, Patricia Santofimia-Castaño, Mircea-Gabriel Stoleriu, Anne Hilgendorff, Stefanie M. Hauck, Ali Ö. Yildirim, Herbert Schiller, Claudia A. Staab-Weijnitz

**Affiliations:** 1 Comprehensive Pneumology Center with the CPC-M bioArchive and Institute of Lung Health and Immunity, Member of the German Center of Lung Research (DZL), Helmholtz-Zentrum München, Munich, Germany; 2 Metabolomics and Proteomics Core, Helmholtz Munich, Munich, Germany; 3 Research Unit Precision Regenerative Medicine, Comprehensive Pneumology Center, Helmholtz Munich, Munich, Germany; 4 Core Facility Statistical Consulting, Helmholtz Munich, Munich, Germany; 5 Laboratory of Respiratory Diseases and Thoracic Surgery (BREATHE), Department Chrometa, KU Leuven, Leuven, Belgium; 6 Department of Pulmonology, Fraunhofer Institute for Toxicology and Experimental Medicine, Hannover, Germany; 7 Pancreatic Cancer, Cancer Research Centre of Marseille, Institute Paoli-Calmettes, Aix-Marseille University, Marseille, France; 8 Institute of Experimental Pneumology, LMU University Hospital, Ludwig-Maximilians University, Munich, Germany; 9 Department of Pediatrics and Division of Pulmonary, Allergy and Critical Care Medicine, School of Medicine, University of Colorado Anschutz Medical Campus, Aurora, CO USA

**Keywords:** NUPR1, Cigarette smoke, Bronchial epithelium, IPF, ALI culture, In vitro, Cell biology, Diseases, Medical research, Molecular biology

## Abstract

The airway epithelium is the first site of injury from cigarette smoke (CS), a major risk factor for chronic lung disease including idiopathic pulmonary fibrosis (IPF). Here, we report the first intracellular proteomic analysis of CS exposure in fully differentiated primary human bronchial epithelial cells (phBECs). Following pathway enrichment analysis, we identified nuclear protein 1 (NUPR1) as a candidate regulator of epithelial stress responses. In contrast to the prediction by pathway enrichment analysis, NUPR1 activity was not altered by CS in vitro. Nevertheless, inhibition of its nuclear translocation using ZZW-115 revealed a cytoprotective and anti-apoptotic role in phBECs, as demonstrated by increased apoptosis and impaired epithelial integrity. *NUPR1* expression was markedly reduced in IPF whole lung tissue and bronchial epithelium. IPF-derived basal cells differentiated into an epithelium exhibiting fewer ciliated and more secretory cells which exhibited significantly higher sensitivity to NUPR1 inhibition. Our findings underscore cell type- and tissue-specific variation in NUPR1-dependent pathways. Collectively, this study positions NUPR1 as a context-dependent epithelial stress regulator whose loss may contribute to epithelial vulnerability in IPF.

**Supplementary Information:**

The online version contains supplementary material available at 10.1038/s41598-026-51510-1.

## Introduction

Idiopathic pulmonary fibrosis (IPF) is a chronic progressive fibrotic interstitial lung disease, which is occurring predominantly in middle aged or older male individuals^1,2^. The disease is characterized by chronic cough, progressive dyspnoea, and ultimately failure of lung function, leading to high mortality rates^1,3^. The median survival is 3–5 years after diagnosis and incidences worldwide are increasing^3^. Although the progression can be slowed down using the anti-fibrotic drugs nintedanib^4^, pirfenidone^5^, and nerandomilast^6^, no curative treatment is yet available. Due to the advanced age of IPF patients and high prevalence of comorbidities, lung transplantations are often contraindicated^1^. Therefore, novel therapeutic options are urgently needed.

While the precise aetiology of IPF remains unknown, cigarette smoke (CS) is increasingly recognized as a major risk factor in its pathogenesis^1,3,7–9^. 41–83% of IPF patients have a smoking history^8,10–12^. Previous studies estimated that adults with a smoking history have a 60% higher lifetime risk to develop IPF^11^. Furthermore, there is a dose- and intensity-dependent correlation between smoking and the risk of IPF development^7^. CS contains more than 4000 chemical compounds and is responsible for the premature death of up to half of active smokers^13,14^. It contributes to the development of various pulmonary diseases, cardiovascular disease and lung cancer^11,15^, causing over 8 million deaths annually^15–17^. This makes smoking one of the most serious threats to public health^14^.

The bronchial epithelium is a pseudostratified layer of cells lining the tracheobronchial tree. It is mainly composed of basal, club, goblet and ciliated cells^13,18,19^. Basal cells act as stem cells of the airway, essential for epithelial regeneration and differentiation into all cell types within the lung epithelium. Ciliated and goblet cells are responsible for mucociliary clearance to remove harmful particles. Mucus is mainly produced by goblet cells. Club cells are the progenitor cells of ciliated and goblet cells. They provide secretory surfactants and uteroglobin (CC10), which has an anti-inflammatory function^18,20^. Moreover, cytochrome P450 enzymes are abundantly expressed in club cells which mediate the detoxification of inhaled xenobiotics^21^. The airway epithelium is the major portal of entry for inhaled toxins, particles and pathogens, including CS, wood dust and herpesvirus infection which are linked to the aetiology of IPF^22,23^. It is well known that the composition of the bronchial epithelium can be altered due to lung disease^18,24^. In recent studies increasing attention has been brought towards the emerging role of the airway epithelium in the pathogenesis of IPF. Most strikingly, a common single nucleotide polymorphism (SNP) in the promoter region of the gene encoding mucin 5B (*MUC5B*) conveys by far the greatest genetic risk to develop IPF^25^. In IPF, *MUC5B* expression is common in distal airway epithelial cells, including transitional zones between bronchioles and alveoli, areas of bronchiolization, and honeycomb cysts, and associates with impaired mucociliary clearance ^26^. But also proximal airways undergo significant remodelling and functional impairment in IPF^24^. However, the mechanisms by which airway epithelial cells and CS exposure contribute to IPF development and progression are incompletely understood.

In the present study, we set out to identify novel CS-induced key regulators in differentiated airway epithelial cells using an unbiased proteomics approach followed by pathway enrichment analysis. This predicted Nuclear protein 1 (NUPR1) as a novel key regulator in these cells which prompted us to study the role of NUPR1 in phBECs including those derived from IPF patients. Our results indicate an important general role for NUPR1 in airway epithelial cell death which is little affected by CS exposure in vitro. Interestingly, differentiated IPF-derived phBECs were significantly more susceptible to NUPR1 inhibition-induced cytotoxicity. Furthermore, after 28 days of differentiation, IPF-derived phBECs were characterized by a marked reduction in ciliated at the expense of goblet cells compared with control phBECs, indicating that IPF-derived basal cells are reprogrammed to yield an airway epithelium with fewer ciliated and more secretory cells.

Some of the results of this study have been previously reported in the form of a conference abstract^27^.

## Materials and methods

For more details on materials and methods, including primers and antibodies used for gene expression analysis (Supplemental Tables S3–S4), see the online supplement. The mass spectrometry proteomics data have been deposited to the ProteomeXchange Consortium via the PRIDE^28^ partner repository with the dataset identifier PXD063763.

### Patient material

Primary human bronchial epithelial cells (phBECs) from three Idiopathic Pulmonary Fibrosis (IPF) and seven non-chronic lung disease (control) donors were provided by the CPC-M bioArchive (Comprehensive Pneumology Center, Helmholtz Munich). The ethics committee of the Ludwig-Maximilians-University Munich approved this study (ethic votes #333-10) and all participants provided a written informed consent. All research was performed in accordance with the relevant guidelines and regulations. PhBECs were isolated as previously described^29–31^. Briefly, bronchi were treated with Pronase E and incubated. Epithelial cells were gently scraped with a scalpel, minced and filtered through a 70 mm strainer to remove tissue. The cells were placed on uncoated petri dishes for 3 h to remove fibroblasts. The supernatant was transferred on a collagen I-coated (136157, ChemCruz) petri dish in Pneumacult Ex-Plus media (5040, Stemcell Technologies) containing 1% penicillin–streptomycin (pen/strep, 15140, Gibco). The cells were tested for *Mycoplasma pneumonia*. The negative tested cells were expanded to passage 1, frozen down and stored in liquid-nitrogen.

One sample of IPF-derived phBECs (donor 7) was obtained from Hannover Medical School. The study was approved by the Institutional Review Boards at Hannover Medical School (approved IRB protocols #2518-2014 and #2923-2015) and University Medical Center Freiburg (approved IRB protocols #239/12 and #03/10). All participants provided a written informed consent. Isolation of phBECs was performed as previously described^32^.

For the NUPR1 inhibition experiment, control phBECs were derived from histologically normal lung regions of patients undergoing lung tumour resections (two males, two females, mean age 73.25 ± 2.29 years (SEM)). IPF phBECs were derived from airways of IPF patients (three males, one female, mean age 65.25 ± 2.17 years (SEM)). For the proteomics analysis, control phBECs were used (three males, one female, mean age 73.50 ± 2.33 years (SEM)). For more details on patient characteristics, please refer to Supplemental Table S1.

Additionally, formalin-fixed, paraffin-embedded (FFPE) tissue samples of peritumoral tissue from five non-chronic lung disease patients were provided by the CPC-M bioArchive (Comprehensive Pneumology Center, Helmholtz Munich). The ethics committee of the Ludwig-Maximilians-University Munich approved this study (ethic votes #19-630) and all participants provided written informed consent. To obtain control FFPE lung sections, fresh lung tissue specimen of approximately 8 mm^3^ were fixed in paraformaldehyde (PFA) for over 24 h at room temperature (RT). Subsequently, they were transferred into PBS at 4 °C before the processing in the tissue processer according to the manufacturer’s instructions and embedding in paraffin. The paraffin blocks were stored at RT prior to sectioning at a thickness of 5 µm.

FFPE lung sections from five IPF patients were obtained from the KU Leuven lung biobank (ethical approval S52174). All participants provided a written informed consent. The FFPE processing was performed as previously described^33^.

For the NUPR1 quantification in lung sections, control FFPE sections were derived from histologically normal lung regions of patients undergoing lung tumour resections (four males, one female, mean age 62.40 ± 5.58 years (SEM)). IPF derived FFPE sections were derived from explanted lungs of IPF patients (four males, one female, mean age 59.80 ± 1.88 years (SEM)). For more details on patient characteristics, please refer to Supplemental Table S2.

### NUPR1 inhibition

N,N-dimethyl-4-[3-[2-(trifluoromethyl)-10H-phenothiazin-10-yl]propyl]-1-piperazineethanamine trihydrochloride (ZZW-115, 34,974 /CAS 10122–45-9, Caymen Chemical) was dissolved in dimethyl sulfoxide (DMSO, D4540/ CAS 67–68-5, Sigma-Aldrich) to prepare a 30 mM stock solution. The stock solution was stored at − 80 °C in aliquots until further use. For NUPR1 inhibition experiments in phBECs the stock solution was diluted in Pneumacult ALI Basal medium (05,041, Stemcell Technologies) to obtain a final concentration of 0–150 µM ZZW-115 and 0.5% DMSO. This inhibition solution was applied to both sides of the transwell membrane for 24 h, with 200 µL added to the apical and 1 mL to the basolateral side.

## Results

### Proteomic analysis of chronically cigarette smoke extract (CSE)-exposed phBECs confirms known CS-regulated pathways and suggests NUPR1 as a potential novel regulator of the airway epithelial response to CS

**Fig. 1:**
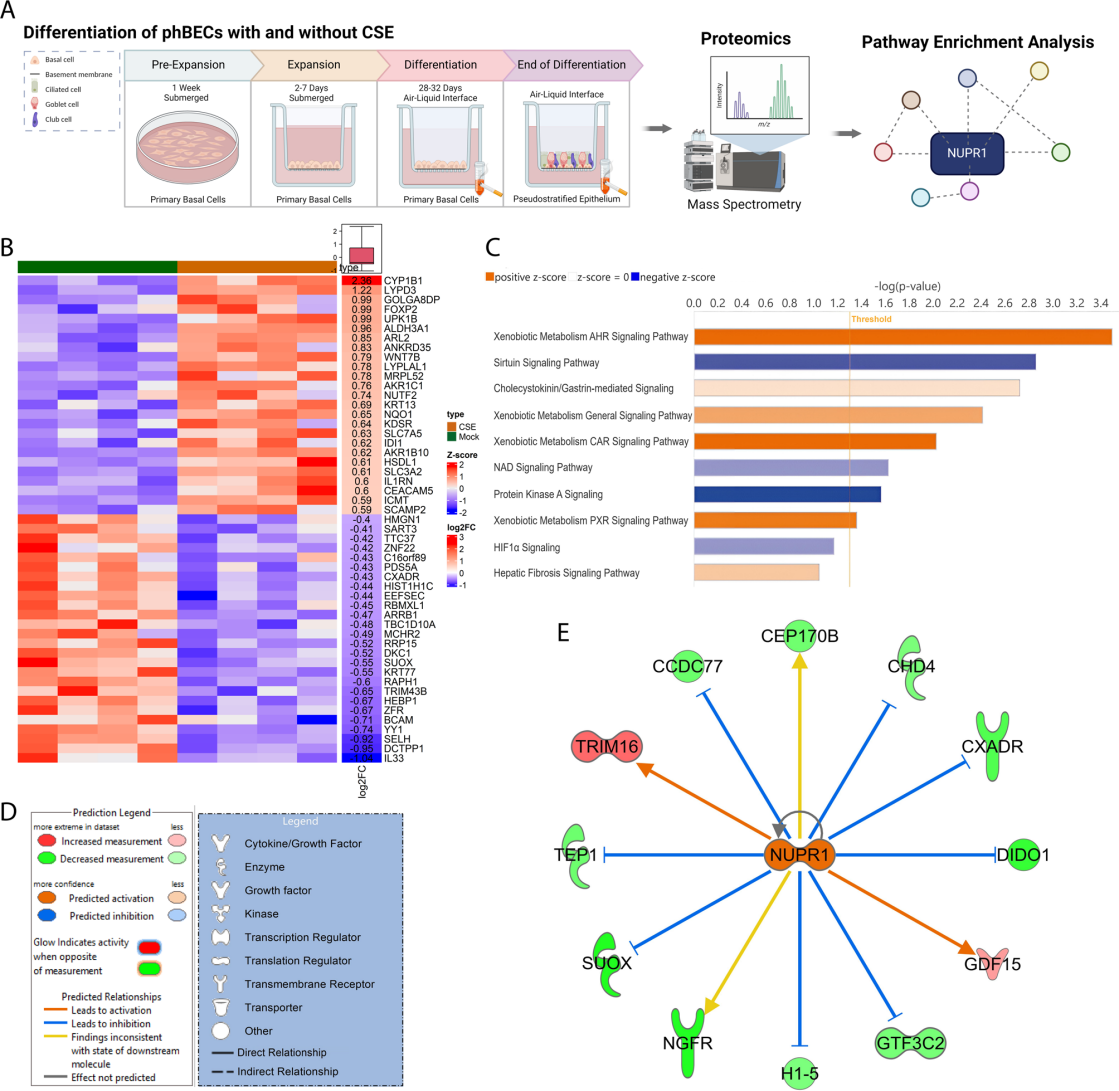
Experimental set-up for proteomic analysis of cigarette smoke (CS)-induced changes in primary human bronchial epithelial cells (phBECs). (**A**) Schematic overview of the differentiation of phBECs, both with and without CS extract at air–liquid interface (ALI). These cells were then submitted to label-free tandem mass spectrometry-based proteomics followed by pathway enrichment analysis(created using Biorender). (**B)** The proteomic data were analysed using a Wald test with Storey’s correction for multiple testing. The heatmap shows protein expression profiles of CS-treated versus control phBECs (pooled time points of all differentiation days for each donor, *n* = 4). It displays the *z*-scores of the top 50 CS-induced differentially expressed proteins by log_2_ fold change (Wald test *q* < 0.05). *Z*-scores were calculated for each protein across the samples to standardize expression levels. Data analysis and visualisation were performed in RStudio. **(C)** Pathway enrichment analysis of the proteomic dataset revealed CS-modulated molecular pathways in phBECs (orange: activation, blue: inhibition; the colour intensity reflects the magnitude of the z-score with darker shades indicating a higher absolute z-value). **(D)** Legend for ingenuity pathway analysis-derived regulatory molecular network (**E**). Proposed CS-activated upstream regulators included **(E)** NUPR1 which is shown in the context of altered target proteins within the data set.

Label-free LC–MS/MS-based proteomic analysis of control phBECs of the pooled data of four time points of differentiation (day 7, 14, 21 and 28, see Fig. 1A) detected in total 4861 distinct proteins, out of which 186 were differentially expressed over the whole time course as a result of chronic CSE-exposure (*q* < 0.05). Overall, the magnitude of expression changes was moderate, with most log_2_ fold changes ranging between 1 and -1. This is in qualitative agreement with our previous study using the same culture and CSE exposure approach where CS-induced changes were much more drastic on the transcript than on the protein level^30^ (Online Supplement 2: “Final Protein List”). The top 50 CSE-regulated proteins according to log_2_ fold changes (Fig. 1B) included, as expected, upregulated enzymes of xenobiotic metabolism (Cytochrome P450 1B1 (CYP1B1), Aldehyde dehydrogenase, dimeric NADP-preferring (ALDH3A1), Aldo–keto reductase family 1 member C1 (AKR1C1), Aldo–keto reductase family 1 member B10 (AKR1B10), NAD(P)H dehydrogenase [quinone] 1 (NQO1)), again confirming our previous findings^30^. Notably, Selenoprotein H (SELH) and Interleukin 33 (IL33) belonged to the most downregulated proteins and using immuno-based approaches, we confirmed loss of intracellular SELH using ELISA and loss of IL33 using Western Blot analysis (Supplemental Fig. S1–S2). While loss of IL33 was found for all assessed time points, loss of SELH was most pronounced at early time points of differentiation.

Next, we subjected this protein list to pathway enrichment analysis to identify potential novel key regulators of the CS response in the airway epithelium. The proposedly altered pathways included such that are well-established to be affected by CS exposure, including activation of the Aryl hydrocarbon receptor (AhR) signalling pathway^34^ and downregulation of Sirtuin signalling pathways^35^ (Fig. 1C). Pathway enrichment analysis suggested 23 key regulators of the CS response (Supplemental Table S5), including known factors like β-catenin^36^, transforming growth factor β1^37^ and KRAS^38^, while NUPR1, to the best of our knowledge, had not been described in the context of the airway epithelial response to CS (Fig. 1D, E, Supplemental Fig. S3). Therefore, we hypothesized that NUPR1 is activated by CS in phBECs and protects from airway epithelial cell death. With airway-like cells emerging as key players in IPF and CS-induced pathways remaining poorly understood in this context, we further investigated whether NUPR1 acts as a survival factor in IPF-derived airway epithelial cells and whether its expression is changed in IPF.

### ZZW-115 efficiently blocks nuclear translocation of NUPR1 and displays dose-dependent cytotoxicity in phBECs

**Fig. 2:**
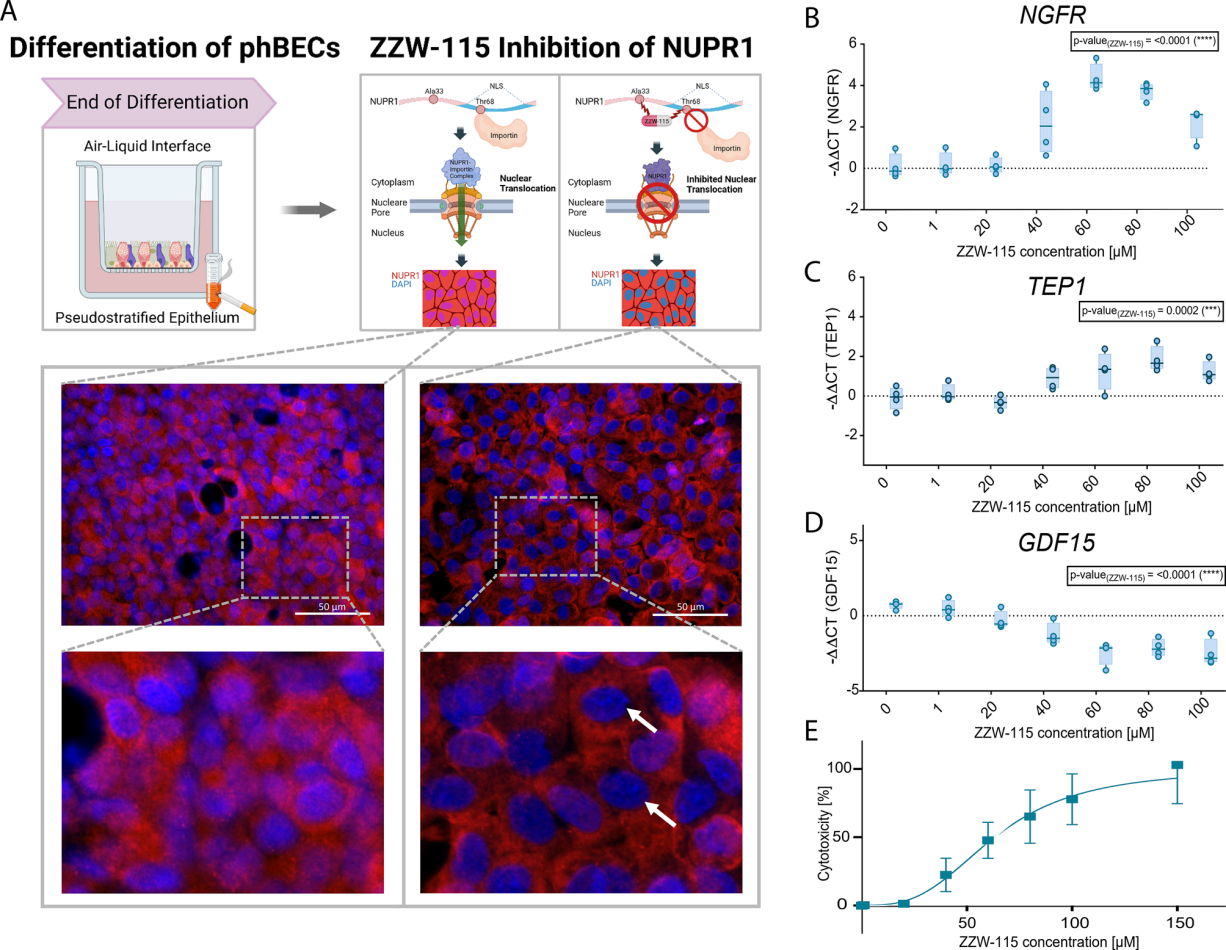
NUPR1 inhibits nuclear translocation in fully differentiated primary human bronchial epithelial cells (phBECs). (**A**) PhBECs were fully differentiated followed by 24 h of NUPR1 inhibition using ZZW-115 (0–150 µM) both on apical and basolateral side. Inhibition of nuclear translocation of NUPR1 was confirmed by immunofluorescence stainings of phBECs treated with DMSO vehicle control (left) and ZZW-115 at a concentration close to its IC_50_ (60 µM, right; scale bar 50 µm; created with Biorender; adapted from^40^). (**B**–**D**) QPCR analysis after 24 h of treatment revealed a significant modulation of IPA-predicted NUPR1-regulated target genes *NGFR, TEP1, GDF15* due to NUPR1 inhibition in a dose-dependent manner (0–100 µM, *n* = 4). (**E**) Lactate dehydrogenase assay revealed that inhibition of NUPR1 by ZZW-115 increased cytotoxicity in fully differentiated control phBECs (*n* = 4) in a dose-dependent manner (IC_50_ = 63.6 µM). All data is presented as mean ± SD.

To study the role of the transcription factor NUPR1 in phBECs, fully differentiated phBECs were treated with the NUPR1 specific inhibitor ZZW-115 (0–150 µM). ZZW-115 prevents the complex formation between NUPR1 and importin, which inhibits the nuclear translocation of NUPR1 and thus NUPR1-induced gene transcription^39^. IF analysis showed strong cytoplasmic retention of NUPR1 protein and a clear reduction of nuclear NUPR1 in treated cells, confirming target engagement in our system (Fig. 2A). Also, NUPR1 inhibition in phBECs confirmed the regulation of selected NUPR1 target genes as suggested by IPA on transcript level (Fig. 2B–D). Finally, ZZW-115 was cytotoxic to phBECs in a dose-dependent manner with an IC_50_-value of 63.6 µM (Fig. 2E). These results validated that ZZW-115 worked efficiently in blocking the nuclear translocation of NUPR1 in our culture model and that inhibition of NUPR1 nuclear translocation induces cell death in phBECs, indicating an important protective role in bronchial epithelial cells.

### *NUPR1* is expressed in basal and ciliated cells

**Fig. 3:**
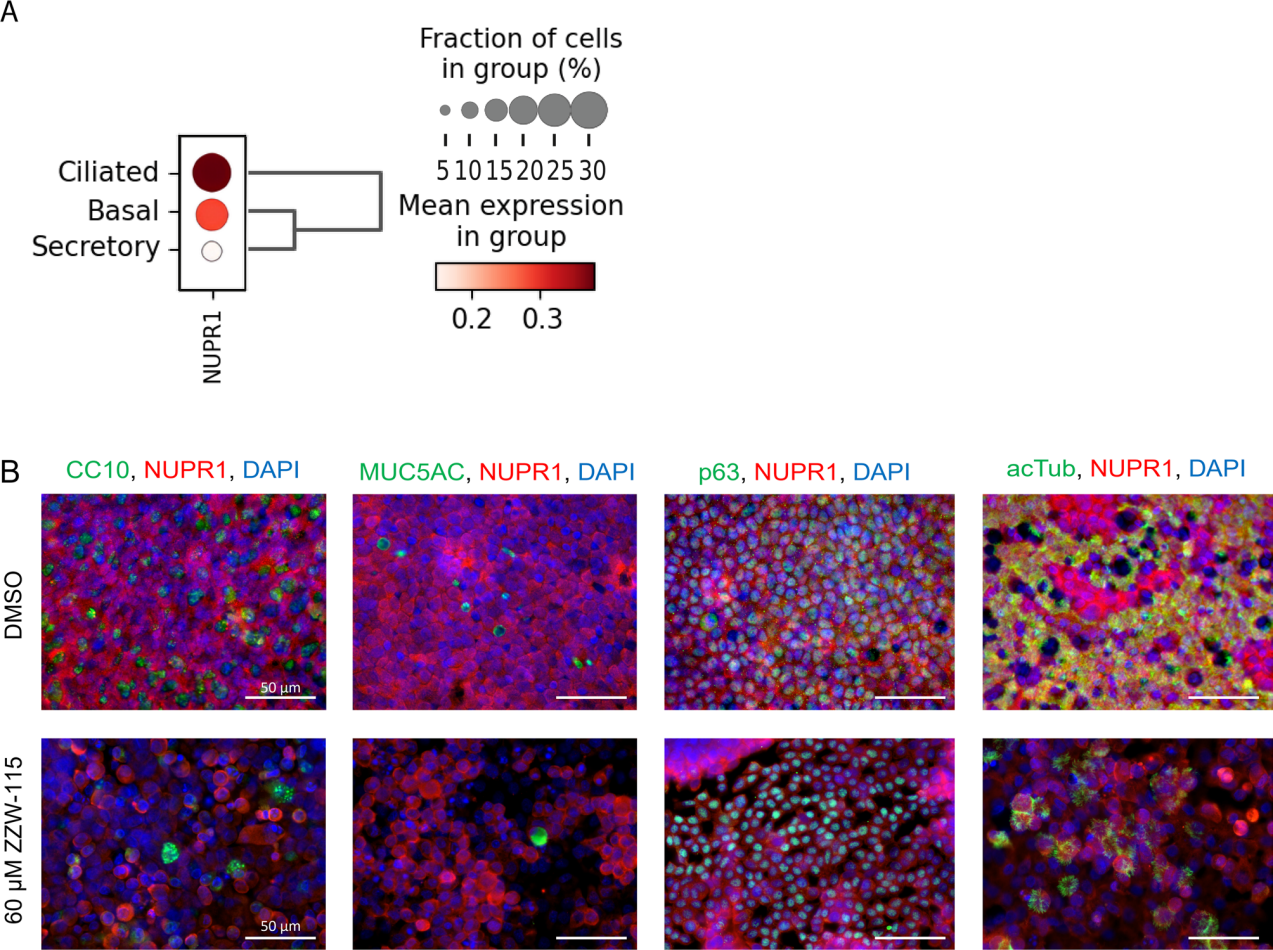
Expression of *NUPR1* in fully differentiated phBECs is restricted to ciliated and basal cells. (**A**) Single-cell RNA sequencing (scRNA-seq) data of fully differentiated control phBECs (*n* = 2) showed that ciliated and basal cells have the highest *NUPR1* expression. (**B**) Representative immunofluorescence of fully differentiated phBECs before and after NUPR1-inhibition with the NUPR1-inhibitor ZZW-115 at its IC_50_ of 60 µM. The co-stainings of NUPR1 with cell type-specific markers for secretory, basal, and ciliated cells (CC10/MUC5AC, secretory cells; p63, basal cells; acTub, ciliated cells) confirmed, that *NUPR1* is expressed in basal and ciliated cells in control (*n* = 4). The stainings after ZZW-115 treatment show cell loss due to cell death after NUPR1-inhibition as well as the inhibition of nuclear translocation of NUPR1 (scale bar 50 µm).

To identify which cells express *NUPR1* in our culture system, we extracted data from an existing scRNA-seq data set from fully differentiated control phBECs^41^. This data set suggested ciliated and basal cells as the main *NUPR1*-expressing cells (Fig. 3A). IF stainings of NUPR1 in combination with cell type-specific markers in control phBECs confirmed the findings of the scRNA-seq analysis in phBECs in vitro, where CC10- and MUC5AC-positive cells, i.e. secretory cells, were always negative for NUPR1 whereas NUPR1-positive cells were positive for p63 (basal cell marker) or acetylated tubulin (ciliated cell marker, Fig. 3B). IF stainings of ZZW-115 treated phBECs showed loss of all cell types due to NUPR1 inhibition induced cytotoxicity (Fig. 3B).

### Cigarette smoke exposure does not activate NUPR1 in phBECs

CS is the most important risk factor for chronic lung disease^42^. Given that IPA analysis of our proteomics study suggested the activation of NUPR1 in phBECs by CS (Fig. 1E) and that NUPR1 has been suggested to fulfil a protective role in alveolar epithelial cells in the context of COPD^43^, we sought to investigate whether CS exposure may activate NUPR1 thus protecting from CS-induced cell death in phBECs.

**Fig. 4:**
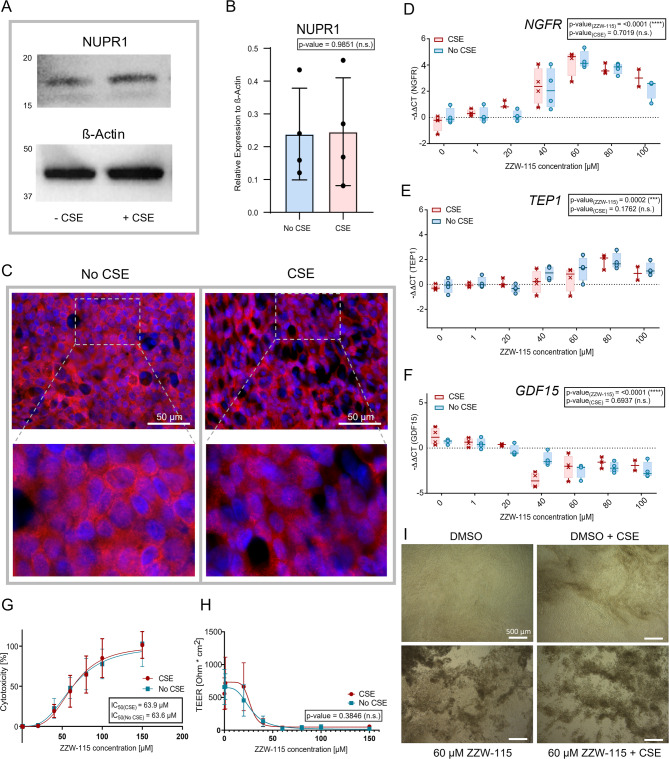
*NUPR1* in fully differentiated phBECs with and without cigarette smoke (CS) exposure. (**A**) Western blot analysis showed no significant difference in NUPR1 protein level in control phBECs + /- CS exposure. ß-actin was used as loading control. Uncropped western blot images are provided in Supplemental Fig. S4. (**B**) Quantification of NUPR1 protein levels relative to ß-actin (*n* = 4). (**C**) Immunofluorescence stainings of untreated control phBECs + /- CS revealed no difference in NUPR1 localization (scale bar 50 µm). (**D**) QPCR analysis of control phBECs (*n* = 4) treated with ZZW-115 (0–100 µM) showed no significant impact on the expression of the IPA- suggested NUPR target genes *NGFR*, (**E**) *TEP1* and (**F**) *GDF15* due to CS exposure. (Data for “No CSE” is identical to the data shown in Fig. 2B, D). (**G**) ZZW-115 increased cytotoxicity and **(H)** decreased cell barrier integrity in fully differentiated phBECs derived from control lungs (*n* = 4) in a dose-dependent manner, without a significant effect due to CS. Cytotoxicity was assessed with a lactate dehydrogenase assay and cell barrier integrity with transepithelial electrical resistance measurements. **(I)** NUPR1 inhibition (60 µM) caused cell loss in control phBECs to a similar extent with and without CS exposure (scale bar 500 µm). [**B** one-way ANOVA followed by Tukey’s post hoc test for multiple comparisons; (**D**–**F**) two-way ANOVA test with multiple comparisons corrected by the two-stage step-up method of Benjamini, Krieger, and Yekutieli; (**G**–**H**) nonlinear regression without weighting was applied and the resulting curves of different conditions (+ /- CS) were compared using an extra sum-of-squares *F*-test]. All data is presented as mean ± SD.

Western blot analysis of fully differentiated control phBECs (*n* = 4) failed to show consistent changes of NUPR1 protein levels in response to CSE exposure (Fig. 4A, B, Supplemental Fig. S4). Additionally, IF stainings did not show any differences in subcellular localization of NUPR1 after CSE treatment (Fig. 4C). QPCR analysis of ZZW-115 treated (0–100 µM) phBECs revealed no changes in transcript levels of selected IPA-predicted NUPR1 target genes (Tumor necrosis factor receptor superfamily member 16* (NGFR), *Telomerase protein component 1* (TEP1), *Growth/differentiation factor 15* (GDF15), *Centrosomal protein of 170 kDa protein B* (CEP170B)*) upon CSE exposure. However, except for CEP170B, these targets were modulated by NUPR1 inhibition in a dose-dependent manner in both conditions (Fig. 4D–F, Supplemental Fig. S5), confirming them as NUPR1-dependent genes in our culture system. As previously shown in Fig. 2, ZZW-115 induced cytotoxicity to phBECs in a dose-dependent manner. However, CSE exposure did not significantly affect the response, resulting in very similar IC_50_ values (63.6 ± 5.1 µM without and 63.9 ± 4.4 µM with CSE; Fig. 4G). In addition, cell barrier integrity was significantly reduced by ZZW-115 treatment to a similar extent with and without CSE (Fig. 4H). Considerable loss of epithelial barrier integrity manifested at comparably lower ZZW-115 concentrations than cytotoxicity, indicating a strong protective effect of NUPR1 in this context. Loss of epithelial barrier integrity was accompanied by considerable ZZW-115-induced cell detachment as visualized by light microscopy (Fig. 4I) and equally apparent in IF stainings (Fig. 3B).

To our surprise, and in contrast to the prediction by IPA, we found no evidence of increased NUPR1 protein levels in cells exposed to CSE. In agreement with these findings, none of the IPA-predicted NUPR1 target genes were changed in their expression upon CSE exposure on transcript level. Finally, prior CSE exposure did not significantly alter cytotoxicity, loss of cell barrier integrity and resulting cell loss induced by NUPR1 inhibition. Collectively, while these findings establish an important role for NUPR1 in bronchial cell viability, they provided no evidence for CS-induced activation of NUPR1 in vitro and thus do not support a specific role for NUPR1 in protecting bronchial epithelial cells from CS exposure-induced cell death.

### Impact of NUPR1 on ferroptosis and apoptosis in phBECs

**Fig. 5:**
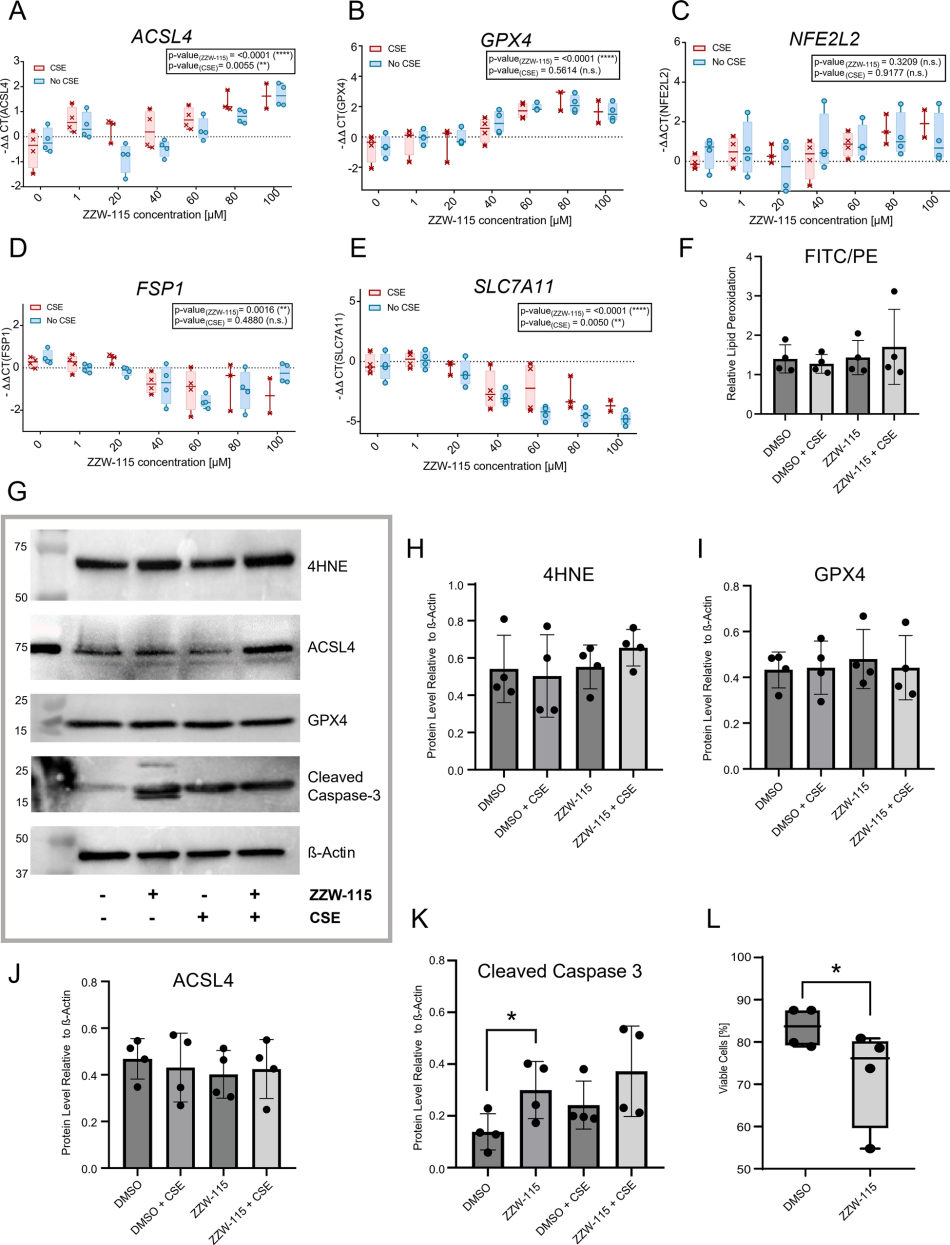
NUPR1 modulation of ferroptosis and apoptosis. (**A**) QPCR analysis of control phBECs (*n* = 4) treated with ZZW-115 (0–100 µM) revealed an increase in *ACSL4*, (**B**) *GPX4* and (**C**) *NFE2L2* and a decrease in (**D**) *FSP1 and* (**E**) *SLC7A11* due to NUPR1 inhibition, with a significant effect of cigarette smoke (CS) on *ACSL4* and *SLC7A11.* (**F**) Lipid peroxidation assay of control phBECs (+ /- CS, *n* = 4) treated with DMSO or ZZW-115 (60 µM) was performed using flow cytometry. The quantification showed no significant modulation in the ratio of the FITC to PE channels, which represent the intracellular lipid peroxidation levels. Consequently, lipid peroxidation was not modulated in any condition. (**G**) Western blot analysis of control phBECs (+ /- CS, *n* = 4) treated with DMSO and ZZW-115 (60 µM) did not show any significant modulation in the protein levels of (**H**) 4HNE-modified protein, (**I**) GPX4 or (**J**) ACSL4, confirming that NUPR1 inhibition does not induce ferroptosis in our culture model. Uncropped images are provided in Supplemental Fig. S7. (**K**) Western blot analysis of control phBECs (+ /- CS, *n* = 4) treated with DMSO or ZZW-115 (60 µM) revealed a significant increase (p = 0.0471(*)) of cleaved caspase 3 on protein levels due to NUPR1 inhibition. However, this effect was not significant in the CS treated condition. (**L**) Flow cytometry of control phBECs (*n* = 4) treated with DMSO or ZZW-115 (60 µM) using Annexin IV/PI showed a significant decrease (*p* = 0.0470) of overall cell viability (Annexin V-negative/PI-negative cells) due to NUPR1 inhibition. [**A**–**E** two-way ANOVA with multiple comparisons corrected by the two-stage step-up method of Benjamini, Krieger, and Yekutieli; **F**, **H**–**L** repeated-measures one-way ANOVA followed by Tukey’s post hoc test for multiple comparisons]. All data is presented as mean ± SD.

Cell death due to NUPR1 loss has been suggested to be induced through ferroptosis^43–46^, apoptosis and necrosis^47–49^, depending on cell type and context. To identify the mechanisms underlying the cytotoxic effects of the NUPR1 inhibition in phBECs, we performed gene expression analysis of ferroptosis-related genes, lipid peroxidation assays, Annexin V/PI apoptosis/necrosis analysis and western blot analysis of ferroptosis and apoptosis-related genes. QPCR analysis revealed significant effects on expression of several ferroptosis-related genes upon NUPR1 inhibition, including an increase in Acyl-CoA synthetase long chain family member 4 *(ACSL4)*, Glutathione peroxidase 4 (*GPX4*) and Nuclear factor erythroid 2-related factor 2 (*NFE2L2)* and a decrease in Ferroptosis suppressor protein 1 *(FSP1)*, Cystine/glutamate transporter *(SLC7A11)* and Heme oxygenase 1 (*HO-1)* expression, while Glutamate-cysteine ligase catalytic subunit *(GCLC)* expression was not modulated (Fig. 5A–E, Supplemental Fig. S6). Of these targets, only *ACSL4* and *SLC7A11* were modulated by CSE. Lipid peroxidation assay with C11-Bodipy 581/591 was performed using flow cytometry. If peroxidation by lipid ROS in cells is occurring, the fluorescence is shifted from PE (reduced) to FITC (oxidized). Our analysis revealed no significant changes in the ratio of the FITC to PE channels, which represent the intracellular lipid peroxidation levels^50^. Consequently, lipid peroxidation levels remained unchanged in control phBECs following ZZW-115 or CSE treatment (Fig. 5F), which was confirmed by western blot analysis for 4-hydroxynonenal (4HNE)-modified protein (Fig. 5G, H, Supplemental Fig. S7), an independent marker of lipid peroxidation. Finally, protein levels of GPX4 and ACSL4 were not altered, neither by NUPR1 inhibition nor by CSE treatment (Fig. 5G, I, J, Supplemental Fig. S7).

Western blot analysis of cleaved caspase 3 showed a significant increase of cleaved caspase 3 and hence apoptosis upon inhibition of NUPR1 without CSE exposure (Fig. 5K, Supplemental Fig. S7). A similar trend was observed in the CSE-treated cells. In agreement with the previously observed cytotoxicity induced by ZZW-115, Annexin V/PI revealed a significant decrease of overall cell viability due to NUPR1 inhibition (Fig. 5L). No significant effect was seen for early apoptosis, late apoptosis or necrosis separately, but a consistent trend towards increase was observed for all three following ZZW-115 treatment (Supplemental Fig. S8). Collectively, these results demonstrate that NUPR1 protects phBECs from cell death in vitro and support a predominantly anti-apoptotic rather than an anti-ferroptotic role in this cell type, which is little affected by prior CSE exposure.

### *NUPR1* expression is lower in whole lung tissue of IPF patients compared to control

**Fig. 6:**
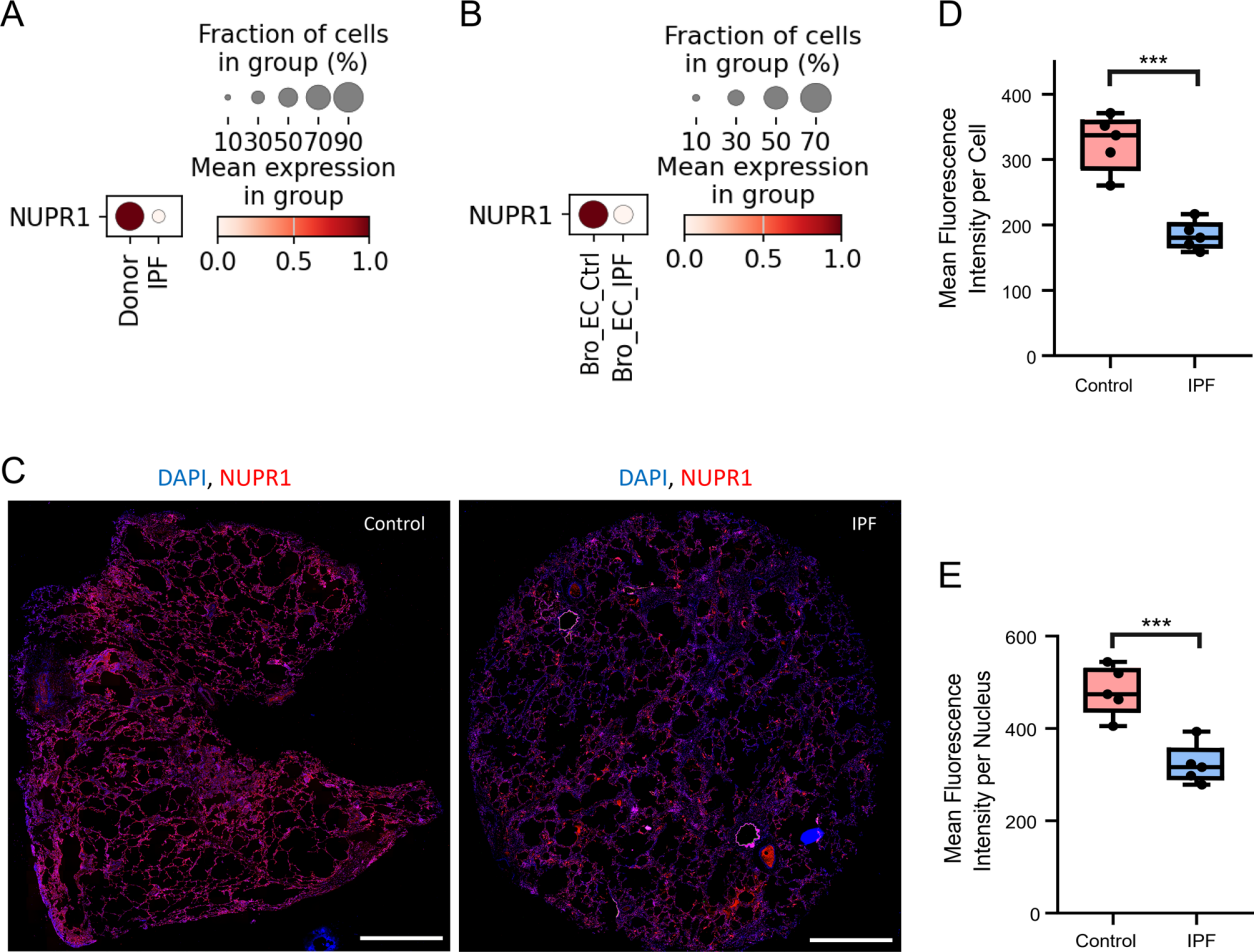
Single cell RNA sequencing (scRNA-seq) and immunofluorescence analysis of NUPR1 in IPF and control samples. (**A**) ScRNA-seq of four combined cohorts showed a higher *NUPR1* expression in IPF compared to control (“Ctrl”) in whole lung and (**B**) bronchial epithelial cells (‘Bro_EC’). (**C**) Representative immunofluorescence stainings of a control and IPF lung sample (scale bar 2000 µm). (**D**) Quantification of NUPR1 mean fluorescence intensity in DAPI-positive cells and (**E**) nuclei of IPF and control lung samples (*n* = 5) revealed a significantly lower expression in IPF samples in both comparisons. The quantification was performed with QuPath 0.5.1. A two-tailed unpaired *t*-test revealed a significantly lower *NUPR1* expression in IPF cells (*p* = 0.0002) and IPF nuclei (*p* = 0.0009) compared to control. All data is presented as mean ± SD.

Airway and airway-like epithelial cells are emerging as key players in IPF aetiology^24^ where the role of NUPR1, to the best of our knowledge, had not been addressed previously. Therefore, we investigated *NUPR1* expression in existing scRNA-seq datasets of four combined control and ILD cohorts (Munich, Chicago, Nashville, New Haven)^24^. This revealed a decrease in *NUPR1* expression in overall lung tissue and also in bronchial epithelial cells in IPF compared to control (Fig. 6A, B). To test these findings, we performed IF stainings in whole lung tissue of IPF and control samples (*n* = 5). These stainings also demonstrated a significant decrease of mean fluorescence intensity and hence *NUPR1* expression in IPF compared to control, both within the whole cells and nuclei (Fig. 6C–E). The scRNA-seq analysis revealed a decreased *NUPR1* expression in all cell types in IPF compared to control (Supplemental Fig. S9A). However, the difference in ciliated cells was marginal. *NUPR1* expression in IPF was highest in ciliated cells followed by basal cells and lowest in secretory cells of IPF-derived phBECs (Supplemental Fig. S9B).

To further assess the impact of CS on NUPR1, we separated *NUPR1* expression in the scRNA-seq dataset according to smoking history. Never and former smokers exhibited significantly higher *NUPR1* expression than active smokers in whole lung, as well as in bronchial epithelial cells (Supplemental Fig. S10). Despite being preliminary, these data indicate that CS exposure may suppress epithelial *NUPR1* expression in bronchial epithelial cells in vivo. Taken together, our findings based on patient lung specimens show that *NUPR1* expression is decreased in IPF and airway epithelial cells compared to control lung tissue and that active smoking decreases *NUPR1* expression. This indicates that chronic environmental exposures may trigger loss of epithelial NUPR1. Exploring whether this acute loss of NUPR1 in airway epithelial cells contributes to lung fibrogenesis is therefore a promising future research direction.

### Following NUPR1 inhibition, IPF-derived phBECs are more susceptible to cell death than control phBECs

**Fig. 7:**
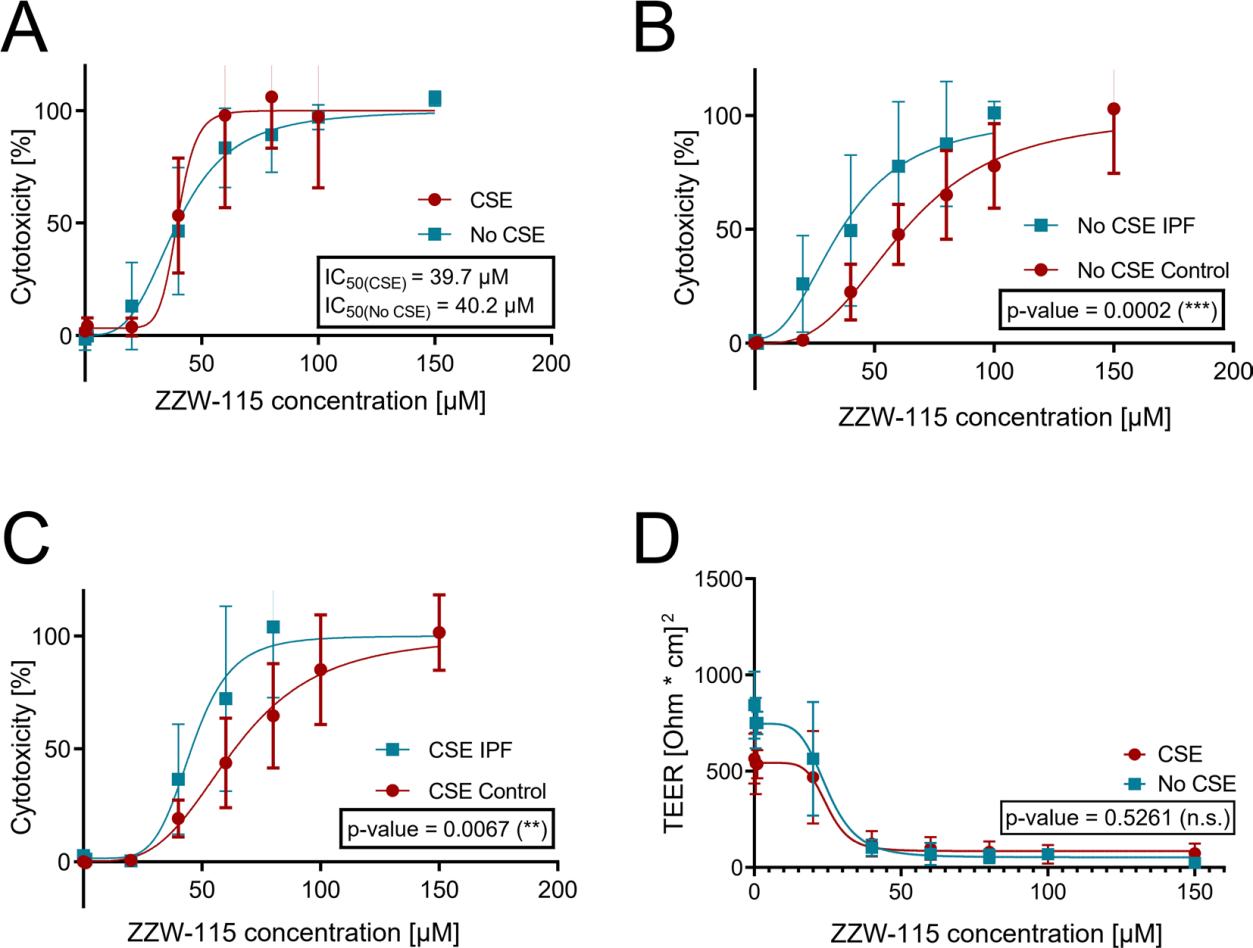
Higher susceptibility to NUPR1 inhibition-induced cytotoxicity in IPF phBECs. (**A**) Lactate dehydrogenase cytotoxicity assay revealed dose-dependent cytotoxicity for the NUPR1 inhibitor ZZW-115 (0–150 µM) in IPF-derived phBECs, both with and without prior CS treatment (*n* = 4). (**B, C**) The IC_50_-values were lower in IPF-derived cells relative to control phBECs, both **(B)** with and **(C)** without CS-treatment compared to control. (**D**) NUPR1 inhibition decreased TEER in fully differentiated phBECs derived from IPF-patients (*n* = 4) in a dose-dependent manner, again unaltered by prior CS exposure. For the analysis of these data, nonlinear regression without weighting was applied and the resulting curves of different conditions (+ /- CS, control vs. IPF) were compared using an extra sum-of-squares F-test. All data is presented as mean ± SD.

Following scRNA-analysis, which suggested a lower *NUPR1* expression in IPF-derived bronchial epithelial cells, we performed analysis in phBECs to investigate whether NUPR1 would protect from bronchial epithelial cell death also in IPF-derived cells. Therefore, cytotoxicity and epithelial barrier integrity were compared in control-derived and IPF-derived phBECs following NUPR1 inhibition by ZZW-115. Similar to control phBECs, NUPR1 inhibition with ZZW-115 increased cytotoxicity in a dose-dependent manner in IPF derived phBECs (*n* = 4, Fig. 7A). However, IPF cells were more susceptible to ZZW-115-induced cell death, displaying a significantly lower IC_50_ value for ZZW-115 relative to control phBECs (40.2 ± 3.6 µM for IPF phBECs vs. 63.6 ± 5.1 µM for control phBECs; Fig. 7B). Similar to our observations in control phBECs, these effects were not significantly modulated by prior CSE exposure of IPF-derived phBECs (39.7 ± 5.1 µM for IPF phBECs vs. 63.9 ± 4.4 µM for control phBECs; Fig. 7C).

Furthermore, also in IPF-derived phBECs cell barrier integrity was significantly impaired in a dose-dependent manner (Fig. 7D), with no significant difference due to CSE exposure. Similar to control phBECs, significant loss of transepithelial electrical resistance occurred at comparably lower ZZW-115 concentrations than cytotoxicity in IPF-derived phBECs, suggesting a protective role of NUPR1 in cell barrier integrity.

### *NUPR1* expression overall is not consistently altered in IPF-derived relative to control phBECs, but IPF-derived bronchial epithelia show fewer ciliated cells

**Fig. 8:**
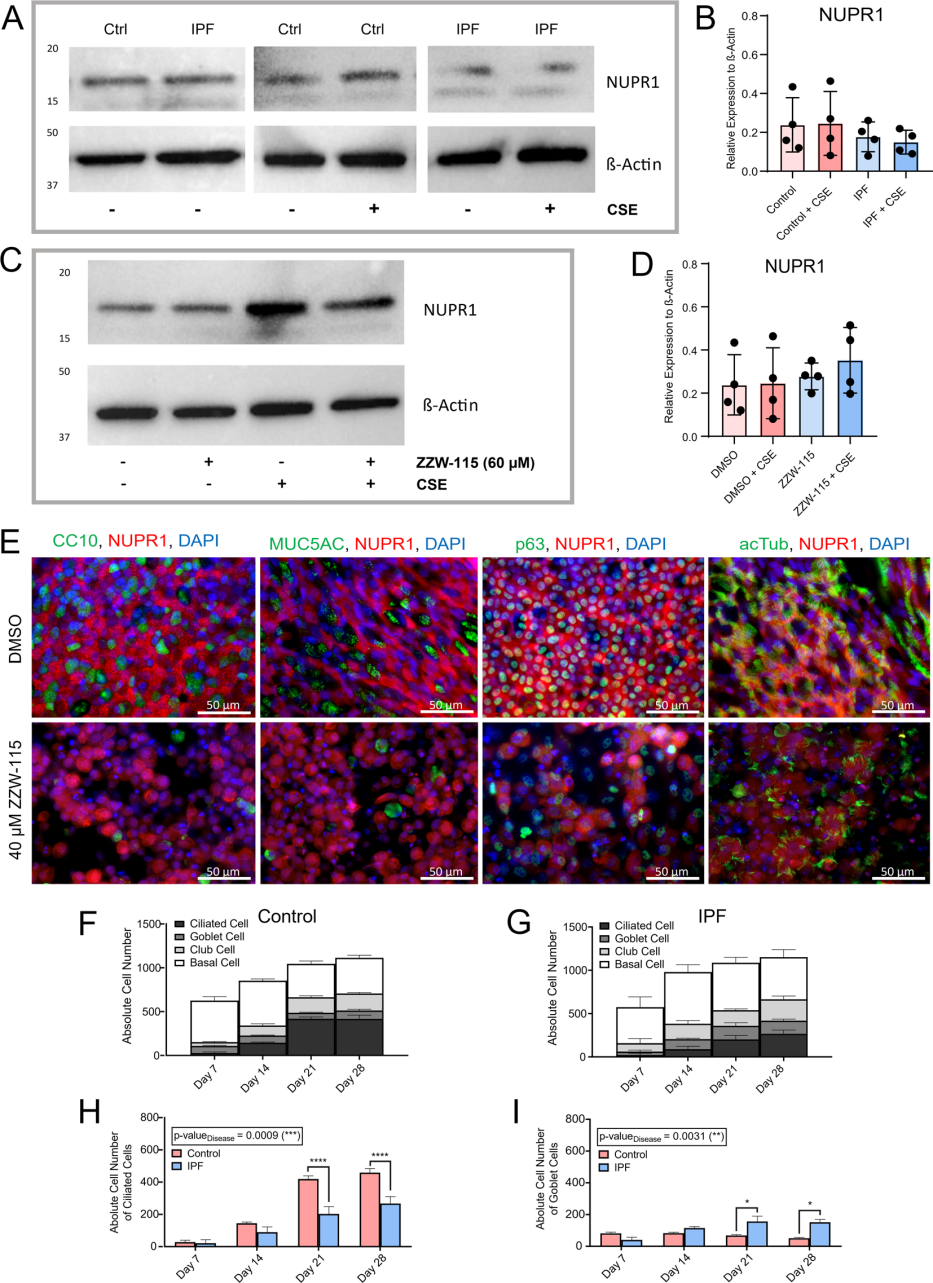
NUPR1 in IPF-derived primary human bronchial epithelial cells (phBECs). (**A**) Western blot analysis of control and IPF-derived phBECs (+ /- cigarette smoke (CS), *n* = 4) did not show any significant difference in the protein levels of NUPR1. ß-actin was used as loading control. (**B**) Quantification of NUPR1 protein levels relative to ß-actin. **(C)** Western blot analysis of control phBECs (+ /- CS, *n* = 4) did not show any significant difference in the protein levels of NUPR1 before and after NUPR1 inhibition. The comparison was conducted between treatments with vehicle control (DMSO) and 60 µM NUPR1-inhibitor ZZW-115 (close to its IC_50_ in control cells). ß-actin was used as loading control. (**D**) Quantification of NUPR1 protein levels relative to ß-actin. (**E**) Representative immunofluorescence of fully differentiated IPF-derived phBECs before and after NUPR1-inhibition with the NUPR1-inhibitor ZZW-115 at its IC_50_ of 40 µM. The co-stainings of NUPR1 with cell type-specific markers for secretory, basal, and ciliated cells (CC10/MUC5AC, secretory cells; p63, basal cells; acTub, ciliated cells) confirmed, that *NUPR1* is expressed in basal and ciliated cells in IPF (*n* = 4). The stainings after ZZW-115 treatment show cell loss due to cell death after NUPR1 inhibition as well as the inhibition of nuclear translocation of NUPR1 (scale bar 50 µm). (**F**) Absolute cell count of the cell type specific quantification of differentiating control and (**G**) IPF-derived phBECs (*n* = 4) at day 7, 14, 21 and 28 at air–liquid interface. Statistical analysis was by two-way ANOVA with multiple comparisons corrected by the two-stage step-up method of Benjamini, Krieger. (**H, I**) Absolute cell type quantification of (**H**) ciliated and (**I**) goblet cells in control and IPF-derived phBECs at day 7, 14, 21 and 28 revealed a significant decrease especially at later timepoints in IPF samples. [**B**, **D** repeated-measures one-way ANOVA followed by Tukey’s post hoc test for multiple comparisons; (**F**–**I**) two-way ANOVA with multiple comparisons corrected by the two-stage step-up method of Benjamini, Krieger]. All data is presented as mean ± SD.

Given that IPF-derived phBECs were more susceptible to NUPR1 inhibition and scRNA-seq analysis suggested a decrease in *NUPR1* expression in IPF compared to control (Fig. 6A, B), we hypothesized that IPF-derived phBECs expressed less *NUPR1* than control phBECs. Western blot analysis revealed very heterogenous protein levels of NUPR1 with a trend towards lower abundance in IPF-derived phBECs which, however, failed to reach significance. Prior CSE exposure again did not alter these protein levels (Fig. 8A–D, Supplemental Fig. S4).

Similar to control phBECs (Fig. 3B), NUPR1 clearly localized to basal and ciliated cells also in IPF-derived phBECs (Fig. 8E), consistent with the findings of the scRNA-seq analysis in IPF-derived bronchial epithelial cells (Supplemental Fig. S9B). To identify the cause of the increased susceptibility of IPF cells to NUPR1-dependent cytotoxicity, the cell populations in both groups were quantified from day 7 to day 28 of differentiation (Fig. 8F, G). While the absolute cell number of NUPR1-positive cells was similar in both groups during the complete course of differentiation (Supplemental Fig. S11A), the analysis of individual cell types revealed a significantly lower number of ciliated cells and an increase in goblet cells in IPF-derived samples (Fig. 8H, I). The quantification of club and basal cells showed a trend towards an increase in IPF-derived cells, which, however, failed to reach significance (Supplemental Fig. S11B–C). Interestingly, this indicates that IPF-derived basal cells are intrinsically primed towards a secretory rather than a ciliated cell fate. Our observations suggest that ciliated cells play a more important role than basal cells in mediating the effects of NUPR1 inhibition in phBECs.

## Discussion

To the best of our knowledge, this is the first study to investigate the intracellular proteomic effects of CS exposure in vitro in fully differentiated phBECs. Whereas several transcriptomic analyses have been reported^51–55^, previous proteomic studies in this context were restricted to extracellular compartments including the secretome or exosomes and have only rarely been performed with primary human cells^56–58^. Here, with the initial aim to identify novel CS-induced key regulators in differentiated phBECs, we performed label-free proteomics followed by pathway enrichment analysis in a validated CS exposure model^30^. The prediction of NUPR1 activation by CS prompted us to study the cytoprotective role of NUPR1 in control and IPF-derived phBECs. While our experimental results did not confirm activation of NUPR1 by CS, our data clearly demonstrate a general protective, predominantly anti-apoptotic, role for NUPR1 in bronchial epithelial cell death in vitro. Notably, NUPR1 levels were lower in IPF whole lung including in bronchial epithelial cells in vivo, and IPF-derived phBECs in vitro were significantly more susceptible to NUPR1 inhibition-induced cytotoxicity.

Pathway enrichment analysis revealed the activation of xenobiotic metabolism and other classical CS-induced pathways in response to chronic CS exposure, overall providing additional validation of our model. Notably, IL33 and SELH were identified as two of the most significantly down-regulated proteins following CS exposure (Fig. 1B, Supplemental Fig. S1–S2). Loss of IL33 is consistent with previous reports linking prolonged CS exposure to decreased *IL33* expression and release^59,60^. This likely reflects a loss of IL33-producing basal cells and may contribute to the regeneration defects upon CSE exposure which we previously described in the same culture system^61^. Loss of SELH, a nuclear redox-regulating protein, in response to CS has not been reported previously and was most prominent at an early differentiation time point (day 7, Supplemental Fig. S1), which aligns with a role of SELH in protecting proliferating cells from oxidative stress^62,63^. Although not the primary focus of this study, these findings warrant the future investigation of IL33 and SELH in the context of stem cell exhaustion and impaired regeneration following CS exposure.

NUPR1 (COM1/p8) is a stress-responsive transcriptional regulator which has been extensively studied in pancreatic disease and cancer^39,44,46,48,64,65^, but comparatively little in the context of chronic lung disease. It has recently been reported that *NUPR1* expression is reduced in a subset of alveolar type II (AT2) epithelial cells in chronic obstructive pulmonary disease (COPD) and downregulated by CSE in the adenocarcinoma cell line A549^43^. Therefore, the predicted activation of NUPR1 by CS in our model was intriguing and given that its role in bronchial epithelial cells remained unexplored, we set out to investigate its function in this context. However, neither NUPR1 protein levels nor nuclear translocation of NUPR1 were changed upon CS exposure in phBECs. While inhibition of NUPR1 by the specific inhibitor ZZW-115 dose-dependently decreased epithelial barrier integrity and induced cytotoxicity, none of these readouts were modulated by CS exposure in vitro*.* Hence, the IPA-derived hypothesis that CS would activate NUPR1 in cultured phBECs was not supported by our follow-up analysis. These findings underscore the importance of experimentally validating computational pathway predictions which frequently are derived from unrelated tissue and molecular contexts. Nevertheless, all tested potential NUPR1-regulated targets were modulated as predicted by IPA in our culture system except for CEP170B, which confirms them as NUPR1-dependent targets also in human bronchial epithelial cells. However, it is important to mention that we cannot exclude the possibility that these findings are indirect and mediated through interactions with other transcription factors that are modulated through NUPR1-inhibition.

As increased cytotoxicity upon loss of NUPR1 has been suggested to be mediated through ferroptosis^43–46^, apoptosis and necrosis^47–49^, depending on cell type and context, we assessed pro- and anti-ferroptotic readouts upon NUPR1 inhibition in our culture system. Even though we observed altered ferroptosis-associated transcript levels upon NUPR1 inhibition in phBECs (Fig. 5A–E, Supplemental Fig. S6), these were not translated to protein level (ACSL4, GPX4) and did not result in altered lipid peroxidation levels. As our CSE exposure did not significantly trigger ferroptosis, a stronger pro-ferroptotic stimulus may be necessary to reveal an anti-ferroptotic role of NUPR1 in phBECs. Instead, our findings support a model where NUPR1 protects bronchial epithelial cells from apoptosis: cleaved caspase 3 was significantly increased after NUPR1 inhibition (Fig. 5K) and, even though early and late apoptosis were not significantly upregulated individually, as monitored by Annexin V/PI, both showed an increasing trend after NUPR1 inhibition (Fig. 5L, Supplemental Fig. S8). Therefore, overall, our results agree with a predominantly anti-apoptotic role rather than an anti-ferroptotic role of NUPR1 in phBECs but at the same time underscore the known context- and cell type-specific nature of NUPR1-dependent protective mechanisms^47,64,66,67^.

Stress-induced airway epithelial cell pathways including apoptosis and increased vulnerability of the airway epithelium are increasingly recognized as an initiating factor in lung fibrogenesis^24,68–70^ but NUPR1 had not been studied in this context. Both scRNA-seq analyses as well as IF stainings in whole lung tissue (Fig. 6A–E) confirmed downregulation of NUPR1 in IPF relative to control lung tissue, including in bronchial epithelial cells. This prompted us to test the hypothesis that IPF-derived phBECs in vitro were more susceptible to NUPR1 inhibition. Notably, phBECs differentiated from IPF-derived basal cells mimicked typical properties of IPF airway epithelium including an increase in secretory cells and a loss of ciliated cells^71,72^, indicating epigenetic reprogramming of airway basal cells that is maintained in culture^68^. Also, the main *NUPR1*-expressing cell types in vitro*, i.e.* basal and ciliated cells, reflected the ones in IPF (Supplemental Fig. S9). Even if loss of NUPR1 protein was not fully recapitulated in IPF-derived *vs*. control phBECs in vitro, IPF-derived cells were indeed considerably more susceptible to NUPR1 inhibition, demonstrating significantly lower IC_50_ values for ZZW-115 (Fig. 7B, C). As NUPR1 expression was at best marginally reduced in IPF-derived phBECs in our study, the increased susceptibility to NUPR1 inhibition likely reflects more nuanced mechanisms. One contributing factor may be differences in cell-type composition, as IPF cultures contained fewer ciliated and more secretory cells (Fig. 8E–I), suggesting that basal and/or secretory progenitor populations may be more vulnerable to NUPR1 inhibition than ciliated cells. Furthermore, IPF-derived epithelial cells are generally more injury-prone due to impaired barrier integrity, elevated ER stress, mitochondrial dysfunction, and increased oxidative stress, all of which reduce resilience to external insults^73–76^. Taken together, these mechanisms may explain the enhanced sensitivity of IPF-derived phBECs to NUPR1 inhibition despite largely preserved NUPR1 expression.

NUPR1 is emerging as a potential target in oncology, including for lung cancer^66,77,78^, because NUPR1 supports survival, therapy resistance, and stress adaptation of tumor cells^64,79,80^; pharmacologic blockade with ZZW-115 suppresses tumour growth and angiogenesis and sensitizes cancer cells to cytotoxic stress^39,49,81^. Our data indicate that NUPR1 is cytoprotective in bronchial epithelium including in IPF-derived cells, raising a safety concern that systemic or airway-exposed NUPR1 inhibitors could cause epithelial cell death and potentially trigger or exacerbate lung fibrosis. Notably, lung cancer and IPF frequently co-occur and share risk factors like age and CS; IPF confers an independently elevated risk of lung cancer and worse outcomes^82–84^. This underscores the need for caution when considering NUPR1 inhibition in cancer patients with fibrotic lung disease.

Our study has several limitations. Our data clearly indicate the involvement of NUPR1 in several mechanisms in phBECs. However, we cannot exclude the possibility that these observed NUPR1-induced effects are indirect and mediated through interactions with other stress-responsive transcription factors or proteins that are modulated via NUPR1-inhibition. Additionally, a conclusive assessment of a protective role from CS exposure-induced cell death would require NUPR1 activation or overexpression experiments. As no specific NUPR1 agonist is currently available and overexpression approaches are technically challenging in fully differentiated phBECs, further studies will be needed to address this question. Moreover, generating fully differentiated phBEC cultures is time-consuming, resource-intensive, and costly, which inherently constrains sample size and experimental depth. Primary cells are known to exhibit a high inter-donor variability and, with an *n* = 4–5, our study may therefore in part have been underpowered to detect some of the CSE-related changes. Additionally, our in vitro model lacks the complex multicellular and immune environment as well as mechanical forces in the lung, which play a crucial role in lung fibrogenesis and fibrosis progression. Future research using co-culture models of phBECs with fibroblasts or immune cells, such as alveolar macrophages, could possibly provide deeper insights into the role of NUPR1. The used culture conditions are generally optimised for cell viability and contain a variety of growth factors, which may influence *NUPR1* expression as well as IPF-specific characteristics, such as the loss of NUPR1. Finally, we used basal cells derived from the proximal bronchi, not distal airway progenitor cells or cells from honeycomb cysts, which are thought to play a more important role in IPF in vivo. Nevertheless, several key observations in our model, including the reduced number of ciliated cells, the increase in secretory cells, and a loss of cellular resilience in IPF-derived phBECs, are consistent with reported epithelial alterations in IPF in vivo, supporting the value of our model as a meaningful platform for mechanistic investigation^71,72^.

## Conclusion

In conclusion, we present the first intracellular proteomic analysis of CS exposure in fully differentiated phBECs, identifying NUPR1 as a candidate regulator of epithelial stress responses. Our results confirm cell type- and tissue-specific differences in NUPR1-dependent pathways and emphasize the need for context-dependent validation of pathway enrichment analysis predictions. Although NUPR1 was not activated by CS in vitro, specific inhibition of NUPR1 nuclear translocation demonstrated that NUPR1 exerts a predominantly anti-apoptotic and cytoprotective role in phBECs. Importantly, NUPR1 is lost in IPF and IPF-derived epithelial cells were significantly more sensitive to NUPR1 inhibition, suggesting that loss of NUPR1-mediated survival mechanisms in the IPF airway epithelium may contribute to disease pathogenesis. Together, these findings position NUPR1 as a context-dependent epithelial stress regulator with potential relevance for epithelial vulnerability in IPF and warrant further investigation into its therapeutic modulation. Furthermore, our study uncovers novel CS-induced reductions in IL33 and SELH, highlighting additional pathways potentially involved in epithelial exhaustion and impaired regeneration.

## Supplementary Information

Below is the link to the electronic supplementary material.

Supplementary Material 1

Supplementary Material 2

## Data Availability

The mass spectrometry proteomics data have been deposited to the ProteomeXchange Consortium via the PRIDE^28^ partner repository with the dataset identifier PXD063763. All other data supporting the findings of this study are available within the paper and its Supplementary Information.
