# Supplementary material for: Nuclear protein 1 is a cell death regulator in primary human airway epithelial cells and reduced in idiopathic pulmonary fibrosis
Source: Sci Rep. 2026 May 11;16:14728. doi: 10.1038/s41598-026-51510-1 (PMC13161242; doi:10.1038/s41598-026-51510-1)
Supplement: Supplementary file 1 — Supplementary Material 1 [file 41598_2026_51510_MOESM1_ESM.pdf]

Nuclear Protein 1 is a Cell Death Regulator in Primary Human Airway Epithelial Cells and Reduced in Idiopathic Pulmonary Fibrosis

Marie Zöller[1], Michal Mastalerz[1], Elisabeth Dick[1], Juliane Merl-Pham[2], Elisabeth Hennen[1], Sai Rama Sridatta Prakki[3], Ashesh Chakraborty[1], Misako Nakayama[1], Markus Klotz[1], Hannah Marchi[3], Ronan LeGleut[4], Laurens J. De Sadeleer[5], Wim A. Wuyts[5], Bart M. Vanaudenaerde[5], Aicha Jeridi[1], Antje Prasse[6], Benedikt Jäger[6], Patricia Santofimia-Castaño[7], Mircea-Gabriel Stoleriu[1], Anne Hilgendorff[1], Stefanie M. Hauck[2], Ali Ö. Yildirim[1,8], Herbert Schiller[3,8] and Claudia A. Staab-Weijnitz[1,9]

ONLINE SUPPLEMENT

Supplemental Material

1. Materials and Methods

Primary Human Bronchial Epithelial Cells (phBECs) Culture Model

PhBECs were seeded, expanded and differentiated as previously described[1-3], with a modification in the differentiation step. For differentiation, 85,000 phBECs at passage 2 were seeded on each collagen IV-coated (C7532, Sigma) 0.9 cm^2 PET membrane insert (353180, Corning, 0.4 µm) of a 12-well plate (352503, Corning).

Transepithelial Electrical Resistance (TEER) Measurement

TEER measurements were performed as previously described[1].

Preparation of Cigarette Smoke Extract (CSE)

Smoke of six research cigarettes (3R4F, Tobacco Health Research, Kentucky) were pumped through 100 mL of Pneumacult ALI Basal medium (05041, Stemcell Technologies) without supplements at a flow rate of 0.3 L/min using an in-house built device. The medium was sterile filtered with a 0.2 µm syringe filter (17761, Sartorius), aliquoted and stored at -80°C. The resulting medium was considered as 100% CSE[1, 4].

Lactate Dehydrogenase (LDH) Cytotoxicity Assay

Apical and basolateral supernatants were collected from each insert. As a positive control, one insert was treated with 2% Triton-X 100/medium for 1 h apical and basolateral. The supernatant of an untreated insert was used as negative control and medium as blank. The release of LDH was analysed with the cytotoxicity detection kit (11644793001, Roche) following the instructions of the manufacturer. Each supernatant was centrifuged for 10 min at 250 g. 5 µL of the supernatant was added with 95 µL medium to the 96-well plate (655101, Greiner) in triplicates. 100 µL of reaction mix was added to each well. The plate was incubated at RT for 30 min protected from light. After incubation, absorbance was measured with a Photometer Elisa Reader CM Sunrise (Tecan).

RNA Isolation, cDNA Synthesis and Real-Time Quantitative Reverse-Transcriptase PCR (qPCR) Analysis

RNA from phBECs was isolated as previously described using RNeasy Plus Mini Kit (74136, Qiagen) according to manufacturer's instructions[1]. The yield and quality were determined with a NanoDrop 1000 Spectrophotometer (Thermo Fisher Scientific). The samples were stored at -80°C until further use.

RNA transcription into cDNA, followed by qPCR analysis were performed as previously described[1]. For qPCR analysis primers were purchased from Eurofins Genomics Germany GmbH (Table S3). The analysis was performed with a Light Cycler 480 II (Roche).

Gene expression changes are reported as -$\Delta\Delta C_t$ and were calculated by normalizing the expression of each target by the reference gene for each condition (ΔC_t (target) = (C_t (target) - C_t (housekeeper gene)) and comparing each condition with the control (-ΔΔCt = -1* (ΔCt (treated sample) - ΔCt (control sample)). DEAH-Box Helicase 8 (*DHX8*) was used as the primary reference gene and Hypoxanthine Guanine Phosphoribosyltransferase (*HPRT*) as second independent reference gene for confirmation of qualitative gene expression changes in all experiments. All results are given with *DHX8* as a reference gene.

Single-Cell RNA Sequencing Analysis (scRNA-seq)

ScRNA-seq data analysis was performed using four combined IPF cohort datasets from Munich[5], Chicago[6], Nashville[7] and New Haven[8]. The raw count matrices for these datasets are publicly available via the Gene Expression Omnibus (GEO) under the following accession numbers: Chicago cohort (GSE122960, Nashville cohort (GSE135893), and New Haven cohort (GSE136831). The dataset for the Munich cohort can be accessed at https://github.com/theislab/2020_Mayr (last accessed 15.07.2025).

From the integrated object, we generated a focused subset by selecting only cells derived from 'Ctrl' (control) and 'IPF' subjects with a recorded smoking status ('Active', 'Former', or 'Never'). This subset consisted of 51324 samples with the following distribution: Ctrl: 12857 active, 3858 former, 26917 never; IPF: 7692 never. As IPF samples lacked ever smokers, *NUPR1* expression analysis by smoking status was limited to non-diseased controls. All downstream analyses were performed using the Scanpy package (v.1.9.5) in Python. Dimensionality reduction and visualization were carried out using Principal Component Analysis (PCA) followed by Uniform Manifold Approximation and Projection (UMAP). Gene expression patterns were visualized using dot plots and matrix plots, highlighting differences across disease states and smoking statuses.

Additionally, pre-existing scRNA-seq data from control and chronic obstructive pulmonary disease-derived phBECs were utilized[9]. For the present study only control phBECs at day 28 of differentiation were included (*n* = 2).

Flow Cytometry Analysis for Apoptosis and Necrosis Detection (Annexin IV/PI)

After 24h of ZZW-115 treatment, phBECs were washed with 1x HBSS (+ Mg^{2+}/Ca^{2+}) before trypsinization. As a positive control, phBECs were treated with 5 µM Staurosporine (HY-15141, MedChemExpress) for 24h both apically and basolaterally. The supernatant, wash and trypsinized cells were pooled and processed for the analysis using the Annexin V Apoptosis Detection Kit APC (eBioscience, 88-8007-74, Thermofisher Scientific) following the instructions of the manufacturer.

The quantification was performed with a FACSymphony A1 flow cytometer (BD Biosciences) and BD FACSDiva software v.6.1.3. FlowJo software v.10.10 (FlowJo LLC) was used for the analysis.

Flow Cytometry Analysis for Lipid Peroxidation Detection (Bodipy 581/591)

After 24h of ZZW-115 treatment, phBECs were washed with 1x HBSS (+ Mg^{2+}/Ca^{2+}) before analysis. As a positive control, cells were treated with 0.5 µM (1S,3R)-RSL3 (19288, Caymen Chemical) for 24h both from the apical and the basolateral side to induce ferroptosis. The treated phBECs were incubated apically with 0.5 mL of a 1:1000 dilution of C11-Bodipy (10257152, Invitrogen) in media for 20 min at 37°C followed by washing and trypsinization. The samples were centrifuged for 5 min at 300 g. The supernatant was discarded, and the cells resuspended in 200 µL of PBS with 0.5% FBS.

The quantification was performed with a FACSymphony A1 flow cytometer (BD Biosciences) and BD FACSDiva software v.6.1.3. FlowJo software v.10.10 (FlowJo LLC) was used for the analysis.

Immunofluorescence Stainings

Immunofluorescence (IF) stainings of phBECs and formalin-fixed, paraffin-embedded (FFPE) tissue sections were performed as previously described[1, 2]. The stainings were imaged with an Axiovert II fluorescent microscope (Carl Zeiss AG). For each phBEC sample, at least three representative images were taken, FFPE sections were imaged in their entirety.

Quantification of FFPE samples was performed using QuPath software v.0.5.1. Mean fluorescence intensity per cell and per nucleus in DAPI-positive cells were calculated for each FFPE section (n = 5) and statistically analysed using GraphPad Prism 9.

Protein Isolation

Protein isolation was performed as previously described[1]. The protein samples were analysed via western blot analysis. For proteomic analysis, both protein and extracellular matrix (ECM) were used. The samples were stored at -20°C until further use.

Western Blotting

The protein concentrations were quantified using Pierce™ BCA Protein Assay Kits (23225, Thermo Scientific) following the manufacturer's instructions. Absorbance was measured with a NanoQuant Infinite M200 Pro (Tecan). Western blot analysis was performed as previously described[1].

Gels were prepared a day before use and stored overnight at 4°C. 10% gel solution contained: 3.35 mL distilled water, 2.1 mL 1.5 M Tris (pH 8.8), 2.75 mL acrylamide (3029.1, Roth), 82.5 µL 10% SDS (AM9822, Invitrogen), 11 µL TEMED, 11 µL ammonium persulfate (APS, A7469, Sigma Aldrich). The 12.5% gel solution contained 2.7 mL distilled water, 2.1 mL 1.5 M Tris (pH 8.8), 3.45 mL acrylamide, 82.5 µL 10% SDS, 11 µL TEMED, 11 µL APS.

The bands were imaged using the Chemidoc XRS+ system (Bio-Rad) and quantified with ImageLab software v.6.0.1 (Bio-Rad) after normalization to β-actin levels. Uncropped images are shown in Supplement Figures S2, S3 and S6. Figure S3 contains three blots that were used for the quantification. To minimize inter-blot variability and allow direct on-blot comparison, control and IPF

samples were loaded together on the same blot. All experimental conditions of each patient (+/- CS, +/- ZZW-115) were always analysed on the same blot. The analysis of normalized NUPR1/ß-actin ratio across all blots indicated no systemic batch effect and showed comparable mean signal intensities between the blots.

Primers and Antibodies

Primers and antibodies used are listed in the supplementary tables (S3-4). The primers were purchased from Eurofins Genomics Germany GmbH.

Enzyme-linked Immunosorbent Assay

Enzyme-linked Immunosorbent Assay (ELISA) of Selenoprotein H (SELH) in phBECs was performed in duplicates using Human Selenoprotein H ELISA Kit (MBS1600039; MyBioSource) according to the instructions of the manufacturer. For this assay protein was isolated from phBECs as described above. Optical density was measured with a Photometer Elisa Reader CM Sunrise (Tecan).

Proteomic Analysis

Proteomic analysis of differentiating phBECs was performed as previously described[10]. Protein lysates and ECM were isolated from phBECs of four donors at different time points of differentiation (day 7, 14, 21 and 28) both with and without CS exposure. The combined samples were submitted to label-free quantitative proteomic analysis via liquid chromatography-tandem mass spectrometry (LC-MS/MS) at the PROT Research Unit Protein Science at Helmholtz Munich. The mass spectrometry proteomics data have been deposited to the ProteomeXchange Consortium via the PRIDE[11] partner repository with the dataset identifier PXD063763.

Ingenuity Pathway Analysis

The MS/MS-based proteomics data of phBECs, both with and without chronic CSE exposure, was used for pathway enrichment analysis. The analysis was performed with Ingenuity Pathway Analysis (IPA, Qiagen). The cut off was set for *q*-values below 0.05 and log fold changes (logFCs) greater 0.3 or lower -0.3, resulting in the identification of significantly altered proteins. These proteins were mapped onto the Ingenuity Pathways Knowledge Base (IPKB), which collects publicly available literature on known protein interactions. These proteins were then ranked to conduct further analyses. IPA was used to identify enriched biological pathways and to construct interaction networks between proteins, master regulators, disease and downstream functions. Additionally, IPA assessed direct and indirect interactions between target genes as well as predicted interactions for genes not included in the proteomic data set.

Statistical Analysis

Differential proteome analysis was performed using R version 4.0.0. The patient effect on protein expression was partially removed using ComBat[12] from the R package sva. A Bayesian linear mixed-effects model (R package blme) was then applied to the corrected data independently for each protein. A weakly informative prior was used for the covariance matrix of the random effects (patients), and non-informative priors were used for the fixed effects (time and CSE). Log fold changes (logFC) were computed as the average effect of CSE over time, and differentially expressed proteins were identified using the Wald test with Storey correction to account for multiple testing (significance threshold $q < 0.05$).

The results are shown as mean ± standard deviation (SD) and were derived from four biological replicates, with each replicate representing a phBECs donor.

For LDH assay and TEER measurements, nonlinear regression without weighting was applied and the resulting curves of different conditions (+/- CSE, control vs. IPF) were compared using an extra sum-of-squares F test. Cell type-specific quantification and qPCR data were analysed using two-way ANOVA with multiple comparisons corrected by the two-stage step-up method of Benjamini, Krieger, and Yekutieli as well as paired two tailed *t*-test. For Western blot analysis, lipid peroxidation assay and Annexin V/PI overall cell viability statistical analysis was performed using repeated-measures one-way ANOVA followed by Tukey's post hoc test for multiple comparisons. Additionally, Annexin V/PI analysis of each individual type of cell death was evaluated using paired two tailed *t*-test. Although non-parametric tests such as the Wilcoxon signed-rank test could be considered here because of the small sample size and possible deviation from normality, we chose a parametric approach because it is more powerful for detecting consistent directional effects and because repeated measures ANOVA explicitly accounts for within-donor variability. Mean fluorescence intensity of IF stainings was compared in IPF and control via a two-tailed unpaired *t*-test.

The applied statistical test can be found in the respective figure legends. The statistical analyses were carried out using GraphPad Prism 9 und RStudio (2022.072+567). Data are reported as statistically significant when *p<0.05, **p<0.01, ***p<0.001, ****p<0.0001.

2. Supplemental Tables

Table S1: Patient Information of phBECs

Donor No.	Diagnosis	Age	Gender	Smoking status	Smoking cessation period in years	Pack years	Experi-ment
**1**	Adenocarcinoma	70	M	Ex-smoker	5	40	1
**2**	Squamous Cell Carcinoma	80	M	Never-smoker	NA	NA	1, 2, 3
**3**	Adenocarcinoma	71	F	Never-smoker	NA	NA	1
**4**	Adenocarcinoma	72	F	Ex-smoker	>20	20-40	1, 3
**5**	IPF	66	M	Ex-smoker	7	40	1
**6**	IPF	61	M	Ex-smoker	10-20	20-40	1
**7**	IPF	63	F	Ex-smoker	>20	4	1
**8**	IPF	71	M	Ex-smoker	>20	60	1
**9**	Histiocytic sarcoma	72	M	Ex-smoker	>20	20-40	2, 3
**10**	Adenocarcinoma	69	M	Ex-smoker	11	20	2
**11**	Adenocarcinoma	73	F	Never-smoker	NA	NA	2, 3
**12**	Adenocarcinoma	65	F	Ex-Smoker	10	40	3

NA – not applicable

List of Experiments:

1 – NUPR1 inhibition experiment

2 – Proteomics analysis

3 – ELISA and WB for Selenoprotein H and Interleukin 33

Table S2: Patient Information of FFPE Lung Sections

Donor No.	Diagnosis	Age	Gender	Smoking status	Smoking cessation period in years	Pack years
**1**	Peritumor	67	F	Ex-smoker	4	46
**2**	Peritumor	73	M	Ex-smoker	5	12.5
**3**	Peritumor	73	M	Ex-smoker	6	48
**4**	Peritumor	45	M	Ex-smoker	13	20
**5**	Peritumor	54	M	Never-smoker	NA	NA
**6**	IPF	60	M	Ex-smoker	17	30
**7**	IPF	57	M	Never-smoker	NA	NA
**8**	IPF	55	M	Ex-smoker	14	22
**9**	IPF	61	M	Ex-smoker	23	10
**10**	IPF	66	F	Ex-smoker	3	40

NA – not applicable

Table S3: Human Primers used for qRT-PCR Analysis

Gene	Forward primer sequence (5'-3')	Reverse primer sequence (5'-3')
ACSL4	GCT ATC TCC TCA GAC ACA CCG A	AGG TGC TCC AAC TCT GCC AGT A
CEP170B	CAG GTG AGC GTG AAG GGT TT	CAT ACA GGG GTG TGC GGT AA
DHX8	TGA CCC AGA GAA GTG GGA GA	ATC TCA AGG TCC TCA TCT TCT TCA
FSP1	GAA TCC CAG TGT GTG AGC GA	GAT GCA CAC CCC TGT GTC AA
GCLC	CGC TTC AGT ACC TTA ACA AG	AAT GGA GAT GGT GTA TTC TT
GDF15	CTA CGA GGA CCT GCT AAC	ACT TCT GGC GTG AGT ATC
GPX4	AAC TTC ACC AAG TTC CTC	GTG GAG CTA GAA ATA GTG G
HPRT	AAG GAC CCC ACG AAG TGT TG	GGC TTT GTA TTT TGC TTT TCC A
HO-1	GGC TTC AAG CTG GTG	AGC TCT TCT GGG AAG TAG AC
NFE2L2	CTT TGT ACT TTG ATG ACT GC	GCC GAA GAA ACC TCA T
NGFR	TCA TCC CTG TCT ATT GCT CCA	TGT TCT GCT TGC AGC TGT TC
NUPR1	GGT CGC ACC AAG AGA GAA GC	CTC CGC AGT CCC GTC TCT AT
SLC7A11	GCT GTC TCC AGG TTA TTC T	GTC TCC AGA GAA GAG CAT TA
TEP1	CTG TGA GCT GCT GTA GTT TC	ACT GAA CCT GAC CGT ACA GGG GTT TTG GGT GTC CTC

Table S4: Antibodies used for Immunofluorescence (IF) Stainings and Western Blot (WB) Analysis

Target	Host	Reference no.	Provider	Dilution	Use
**αTUB**	Rabbit	ab179484	Abcam	1:500	IF
**CC10**	Mouse	sc365992	Santa Cruz	1:300	IF
**MUC5AC**	Mouse	ab3649	Abcam	1:250	IF
**NUPR1**	Rabbit	T274	Homemade*	1:400	IF, WB
**p63**	Mouse	ab735	Abcam	1:100	IF
**Donkey anti-mouse (red 568)**	Donkey	A10037	Invitrogen	1:400	IF
**Donkey anti-rabbit (red 568)**	Donkey	A10042	Invitrogen	1:400	IF
**Goat anti-mouse (green 488)**	Goat	F2761	Invitrogen	1:400	IF
**Goat anti-rabbit (green 488)**	Goat	A11008	Invitrogen	1:400	IF
**4HNE**	rabbit	ab46545	Abcam	1:1000	WB
**ACSL4**	rabbit	sc-271800	Santa Cruz Biotechnology	1:250	WB
**Caspase3-cleaved**	rabbit	9664S	Cell Signaling Technology	1:1000	WB
**GPX4**	rabbit	ab125066	Abcam	1:1000	WB
**IL33**	goat	AF3625	R&D Systems	1:1000	WB
**β-Actin (13E5) (HRP Conjugate)**	rabbit	5125S	Cell Signaling Technology	1:1000	WB
**Anti-rabbit IgG, HRP-linked Antibody**	rabbit	7074S	Cell Signaling Technology	1:3500	WB

*Kindly provided by Patricia Santofimia-Castaño (Cancer Research Centre of Marseille) (Lan et al. 2020; Santofimia-Castaño et al. 2024).

Table S5: Upstream Regulators Predicted by Ingenuity Pathway Analysis of phBECs

Upstream Regulator	Molecule Type	Predicted Activation State	Activation *z*-score	*p*-value of overlap	Target Molecules in Dataset
**beta-estradiol**	chemical - endogenous mammalian	Activated	2,203	0,0004	*ABCB11, AGO2, APEX1, AQP5, CALM1, CAPN13, CXADR, CYB561, CYP1B1, F3, FOSL1, G6PD, GCLC, GDF15, GSTM3, H6PD, HMGCS1, IPO4, KRT13, LARP6, MAP2K1, MRPL52, MUC5B, MX1, NGFR, NME3, NPC1, PLEKHA6, PRC1, PRKCB, PXMP4, RAC2, RGS9, S100A9, SBF1, SEMA3A, SLC3A2, SLC7A5, SLPI, STS, TIMP3, TNC, TRIM43/TRIM43B, WNT7B, YY1, ZNF22*
**aflatoxin B1**	chemical - endogenous non-mammalian	Activated	2,72	0,000367	*ALDH3A1, GCLC, GDF15, GSTM3, IDI1, MTHFD1L, NQO1, PRC1, SULF2, TRIM16, UGT1A6*
**PD98059**	chemical - kinase inhibitor	Inhibited	-2,422	0,0112	*ARRB1, COL4A1, CYP1B1, F3, FOSL1, GRN, IL1RN, MUC5B, NPC1, NQO1, RETSAT, S100A8, SLC3A2*
**EDN1**	cytokine	Activated	2,416	0,00184	*ANXA6, COL4A1, FOSL1, GRN, MAP2K1, PRKCB, TIMP3, TNC*
**KRAS**	enzyme	Activated	2,436	7,79E-12	*AGO2, AKR1B10, AQP5, BCAM, BZW2, CAVIN1, COL4A1, CXADR, DPYSL2, F3, FOSL1, G6PD, GCLC, GRN, GSTM3, H1-2, HMGA1, IL33, MAP2K1, MX1, NDE1, NPC1, NQO1, NUTF2, PCDH1, PI4KA, PRC1, PYCARD, RHOB, SERBP1, SLC3A2, STEAP4, TIMP3, TNC, YY1*
**GSR**	enzyme	Inhibited	-2	0,000582	*GCLC, ME1, NQO1, TKT*
**TGFB1**	growth factor	Activated	2,438	0,0000011	*ADAM15, AKR1C1/AKR1C2, BUD31, CAB39, CALM1, CEACAM5, CHD4, COL4A1, CRIP2, CXADR, CYB561, DKC1,*

					DPYSL2, F3, FOSL1, FUS, GCLC, GDF15, H1-10, H6PD, HEBP1, HLA-DPA1, HMGA1, HMGCS1, IDI1, IL1RN, IL33, KDELR2, KRT14, LEMD3, MAP2K1, ME1, MLXIP, MUC5B, PCDH1, PRC1, PYCARD, RHOB, SEMA3A, SLC3A2, SLC7A5, SRM, TAX1BP3, TIMP3, TNC, WNT7B
**AKT1**	kinase	Activated	2,789	0,00165	*AKR1B10, AKR1C1/AKR1C2, COL4A1, GCLC, GDF15, HMGCS1, ME1, NQO1, RHOB, SLC3A2*
**AR**	ligand-dependent nuclear receptor	Activated	2,164	0,00535	*AKR1C3, ANXA9, DCTPP1, GDF15, HDAC3, KDELR2, KRT14, NGFR, NPC1, PDIA5, PSCA, RHOB, SLC3A2, SULF2, TIMP3, XPOT*
**NR1I2**	ligand-dependent nuclear receptor	Activated	2,219	0,0186	*CES1, GSTM3, HMGCS1, IL33, S100A8, UGT1A6*
**PPARG**	ligand-dependent nuclear receptor	Activated	2,449	0,0245	*CEACAM5, CES1, FOSL1, GDF15, H6PD, HEBP1, IL33, ME1, RHOB, S100A8, TKT*
**INSIG1**	other	Inhibited	-2,236	0,00723	*G6PD, HMGCS1, IDI1, S100A8, S100A9*
**F2**	peptidase	Activated	2,17	0,0104	*ABCB11, COL4A1, F3, FOSL1, HMGA1, RAC2, RHOB, SRM*
**GLIS1**	transcription regulator	Activated	2,216	0,0000393	*ALDH3A1, COL4A1, CYP1B1, S100A9, TIMP3*
**NFE2L2**	transcription regulator	Activated	2,694	0,000761	*ABCB11, AKR1B10, AKR1C1/AKR1C2, ALDH3A1, G6PD, GCLC, IL1RN, LGALS8, ME1, NQO1, PHKA1, PRKCB, TKT, UGT1A6*
**NUPR1**	transcription regulator	Activated	2,309	0,0104	*CCDC77, CEP170B, CHD4, CXADR, DIDO1, GDF15, GTF3C2, H1-5, NGFR, SUOX, TEP1, TRIM16*

**CTNNB1**	transcription regulator	Activated	2,699	0,0151	*ALDH1A3, AQP5, CALM1, COL4A1, CYP1B1, FOSL1, GDF15, HDAC3, HMGCS1, IDI1, MAML1, ME1, NGFR, NME3, RPS4Y1, S100A8, TIMP3, TNC, WNT7B*
**FOXA2**	transcription regulator	Activated	2,425	0,0493	*CYB561, GSTM3, IL33, MUC5B, SPEF2, WNT7B*
**BACH1**	transcription regulator	Inhibited	-2,219	0,000768	*CALM1, GCLC, ME1, NDUFA6, NQO1*
**IKZF1**	transcription regulator	Inhibited	-2,16	0,0102	*G6PD, GRN, IFIT5, MLXIP, NGFR, PRRT3, SLPI, SULF2*
**FOXO4**	transcription regulator	Inhibited	-2	0,0355	*COL4A1, HMGCS1, IDI1, LEMD3*
**STAT5B**	transcription regulator	Inhibited	-2,121	0,0429	*AKR1C1/AKR1C2, AKR1C3, CASP6, ME1, NQO1, SERPINB6, SLC39A10, UBE2L6*
**UCP1**	transporter	Activated	2,607	0,00201	*BZW2, G6PD, GDF15, MTHFD1L, SLC3A2, SRM, XPOT*

3. Supplemental Figures

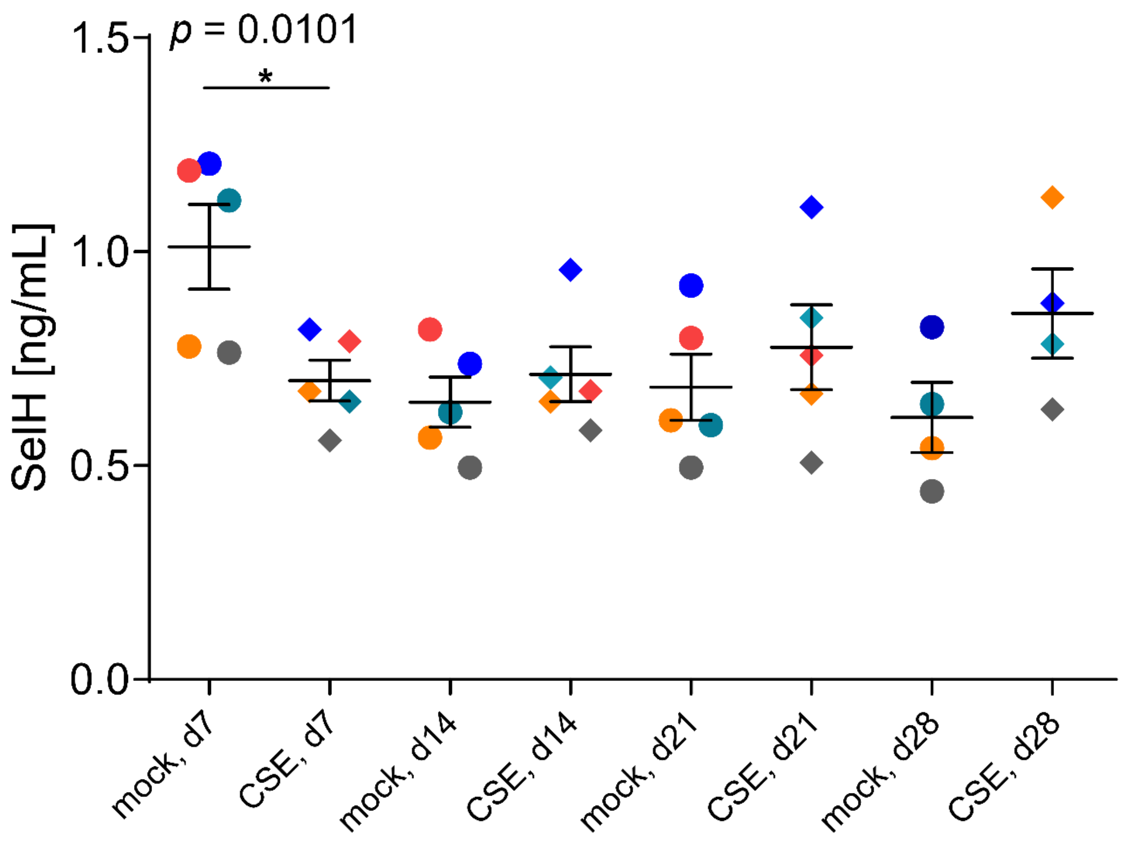

Supplemental Fig. S1: Cigarette smoke (CS) induces loss of intracellular Selenoprotein H (SELH) in primary human bronchial epithelial cells (phBECs). ELISA assay was performed at four timepoints of differentiation (day 7, 14, 21, 28) in cultures with and without cigarette smoke extract (CSE). Data presented as mean ± SD (n = 5). The statistical analysis with a two-tailed paired t-test revealed a significant decrease of intracellular SELH at early timepoint. All data is presented as mean ± SD.

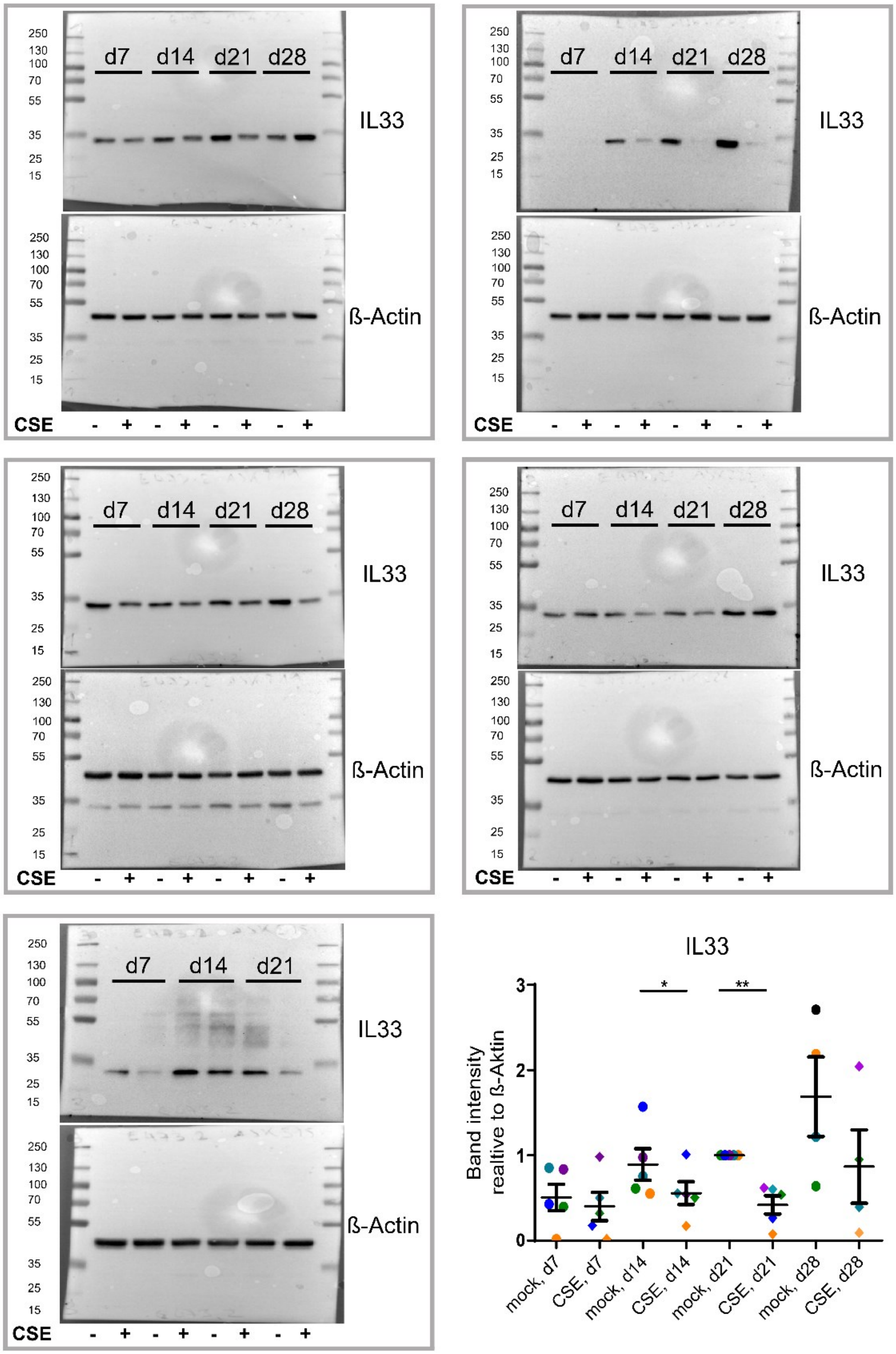

Supplemental Fig. S2: Western blot analysis of IL33 in phBECs with and without CS exposure. Uncropped blots of five donors at 4 timepoints of differentiation (day 7, 14, 21, 28) including the loading control ß-Actin. Data for day 28 of the last donor is unavailable due to insufficient protein yield. Data presented as mean ± SD (n = 5). The statistical analysis with a two-tailed paired *t*-test revealed a significant decrease of intracellular IL33 at day 14 and 21. All data is presented as mean ± SD. Faint bands in ß-Actin blots (e.g., middle panel, left) correspond to remaining signal from IL33.

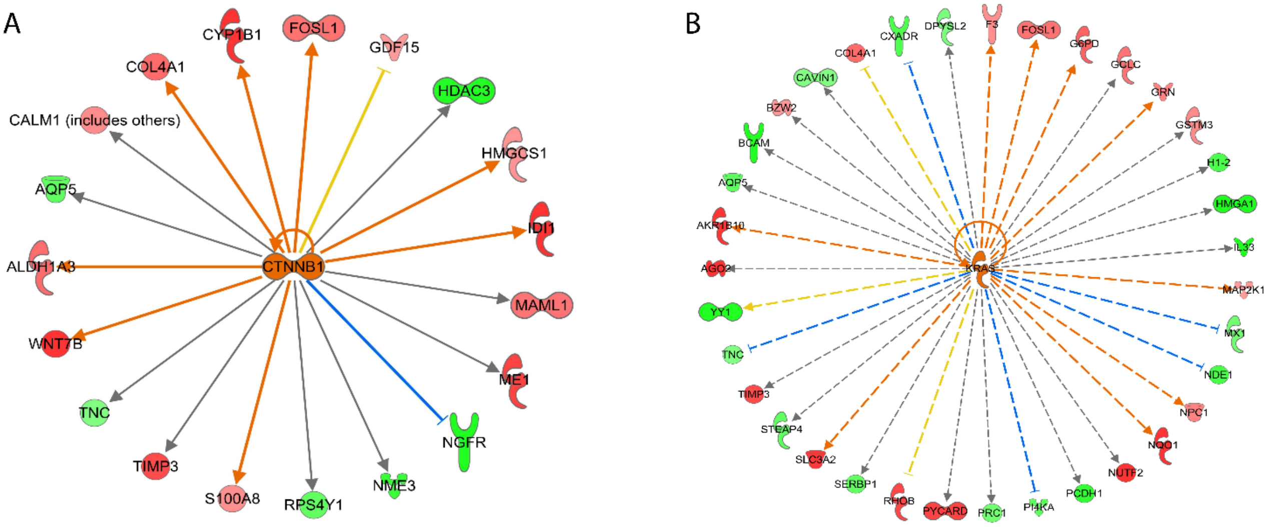

Supplemental Fig. S3: Cigarette smoke activated upstream regulators (A) β-catenin and (B) KRAS in the context of altered target proteins suggested by pathway enrichment analysis of proteomic dataset. The schematic overview of the experiment, statistical analysis of the proteomic data and the legend for ingenuity pathway analysis-derived regulatory molecular networks are shown in Fig. 1 of the main manuscript.

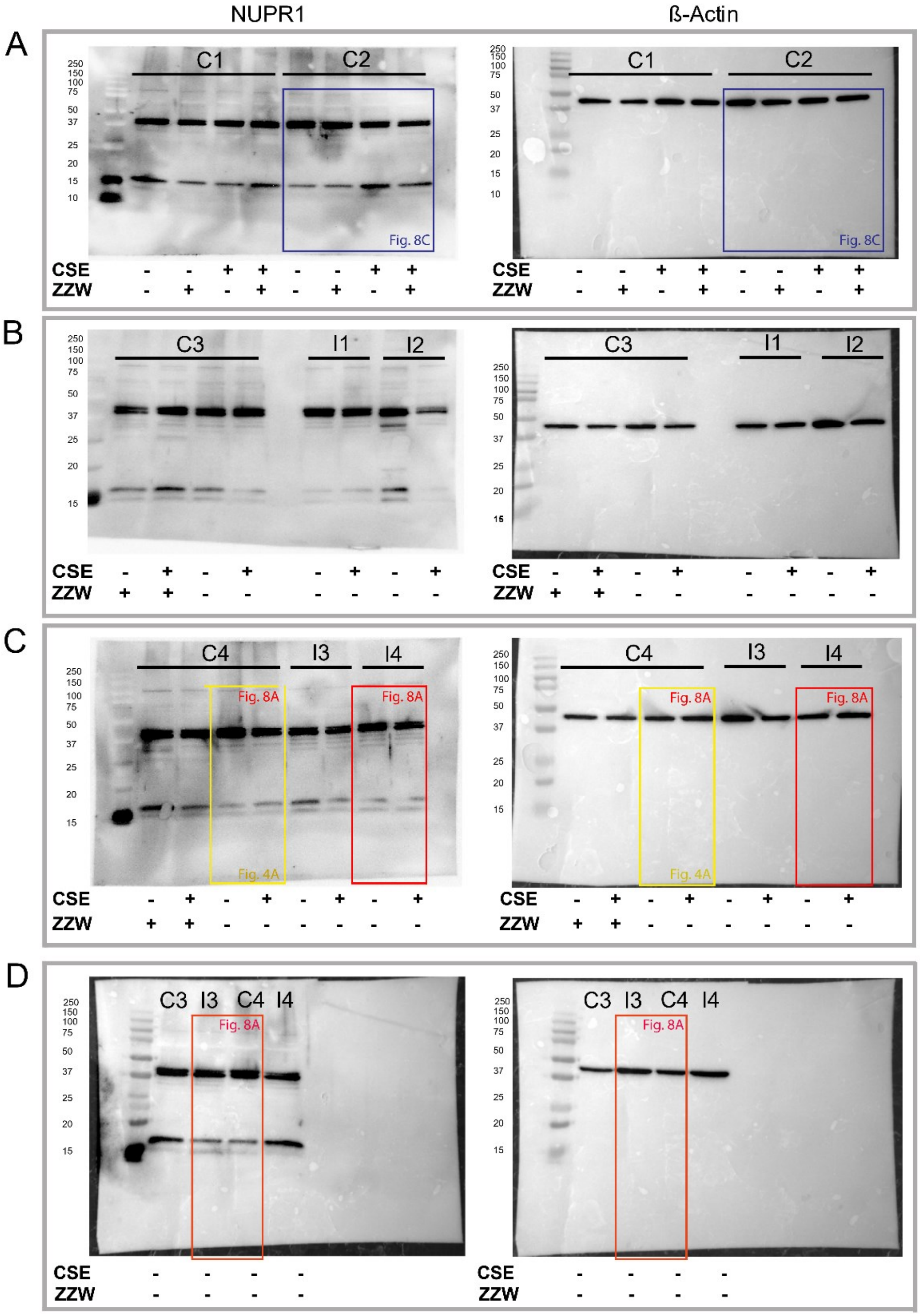

Supplemental Fig. S4: Western blot analysis of NUPR1. Uncropped immunoblots of fully differentiated control (C1-4) and IPF-derived (I1-4) phBECs (*n* = 4). The blots compare control and IPF, CS-treated ('CSE') and untreated cells, as well as vehicle control (DMSO) and NUPR1-inhibition treatment with 60 µM ZZW-115 ('ZZW'). ß-actin was used as loading control. The yellow boxes indicate the cropped regions shown in Fig. 4A, the red and blue boxes represent the cropped regions displayed in Fig. 8A and 8C. Panels **A-C** were used for the quantifications shown in Fig. 8, panel **D** provides an additional representative comparison of IPF- vs control-derived samples.

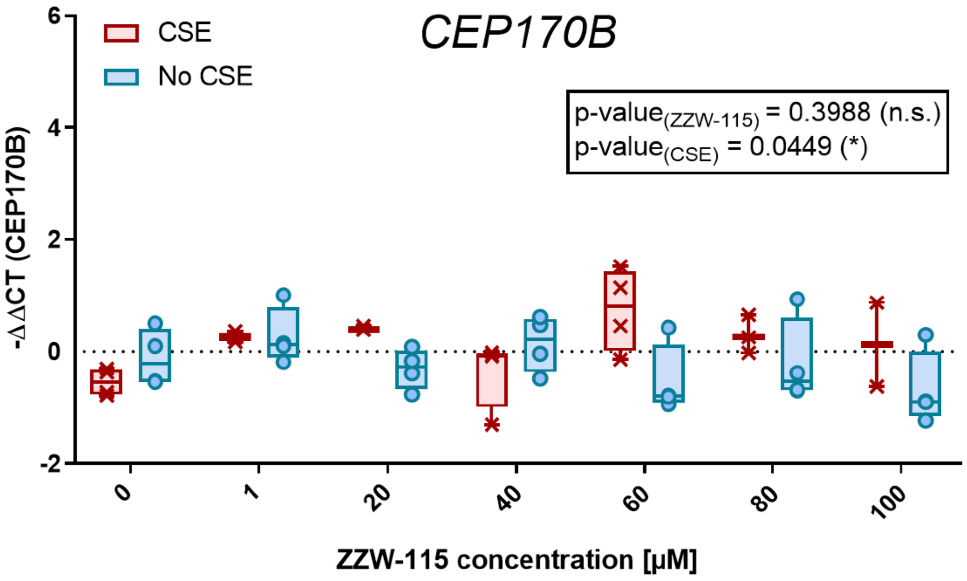

Supplemental Fig. S5: QPCR analysis of *CEP170B* in control phBECs (*n* = 4). NUPR1 inhibition with ZZW-115 (0-100 µM) as well as cigarette smoke treatment did not modulate the target. The statistical analysis was performed in comparison to the vehicle control (DMSO) using a two-way ANOVA test with multiple comparisons corrected by two-stage step-up method of Benjamini, Krieger, and Yekutieli. All data is presented as mean ± SD.

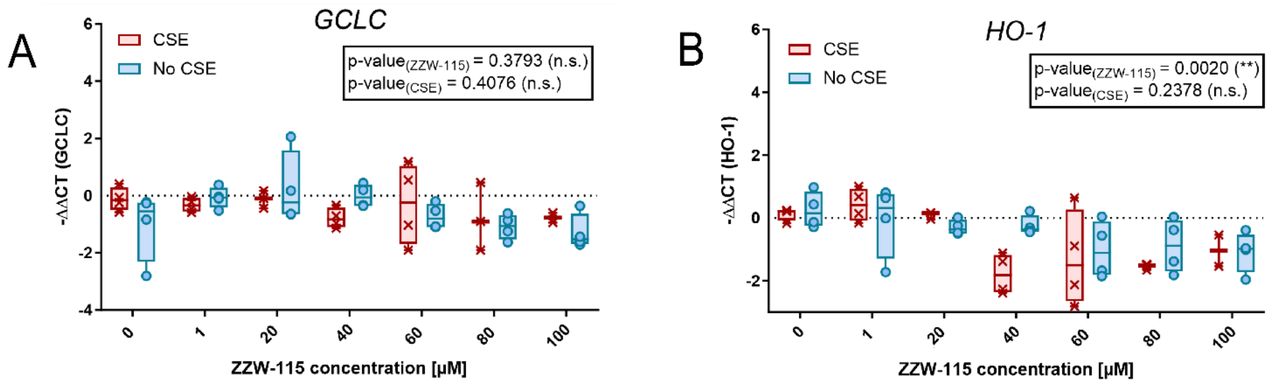

Supplemental Fig. S6: QPCR analysis of *GCLC* and *HO-1* in control phBECs (*n* = 4). (A) NUPR1 inhibition with ZZW-115 (0-100 µM) as well as cigarette smoke treatment did not modulate the GCLC. **(B)** The expression of *HO-1* was not modulated by cigarette smoke but was decreased due to NUPR1 inhibition. The statistical analysis was performed in comparison to the vehicle control (DMSO) using a two-way ANOVA test with multiple comparisons corrected by two-stage step-up method of Benjamini, Krieger, and Yekutieli. All data is presented as mean ± SD.

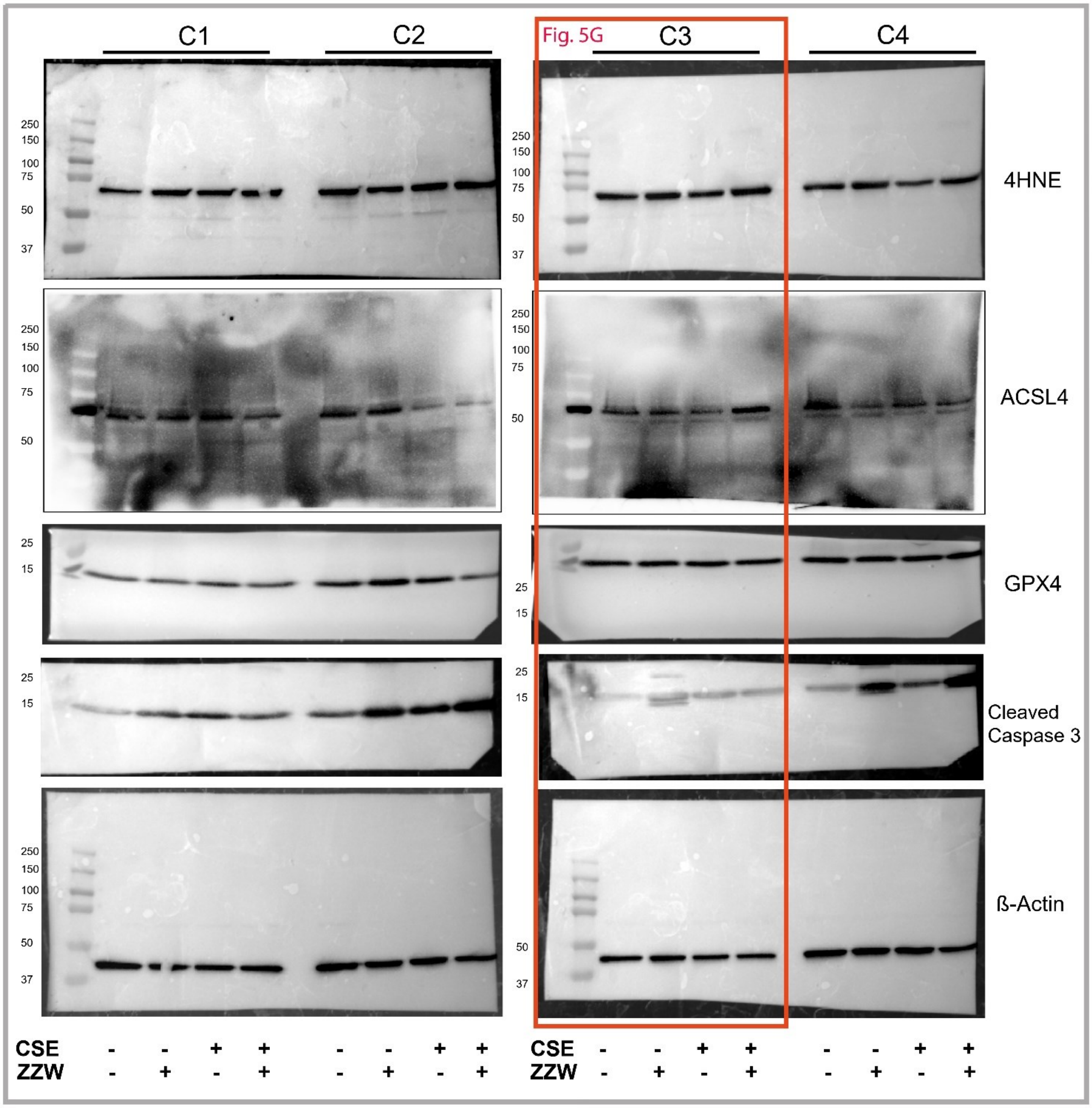

Supplemental Fig. S7: Western blot analysis of 4HNE, ACSL4, GPX4 and cleaved caspase 3 in control phBECs (*n* = 4). The immunoblots compare the influence of CS-treated and untreated cells, as well as vehicle control (DMSO) and NUPR1-inhibition treatment with 60 µM ZZW-115 on these targets. 4HNE, ACSL4 and GPX4 were not significantly modulated in any treatment condition. Cleaved caspase 3 was significantly upregulated due to ZZW-115 treatment compared to DMSO, but only in absence of cigarette smoke. ß-actin was used as loading control. The red box indicates the cropped regions shown in Fig. 5G. Membranes were cut between the 37 and 25 kDa markers to avoid repeated stripping and reprobing of the same membrane and the associated loss of signal.

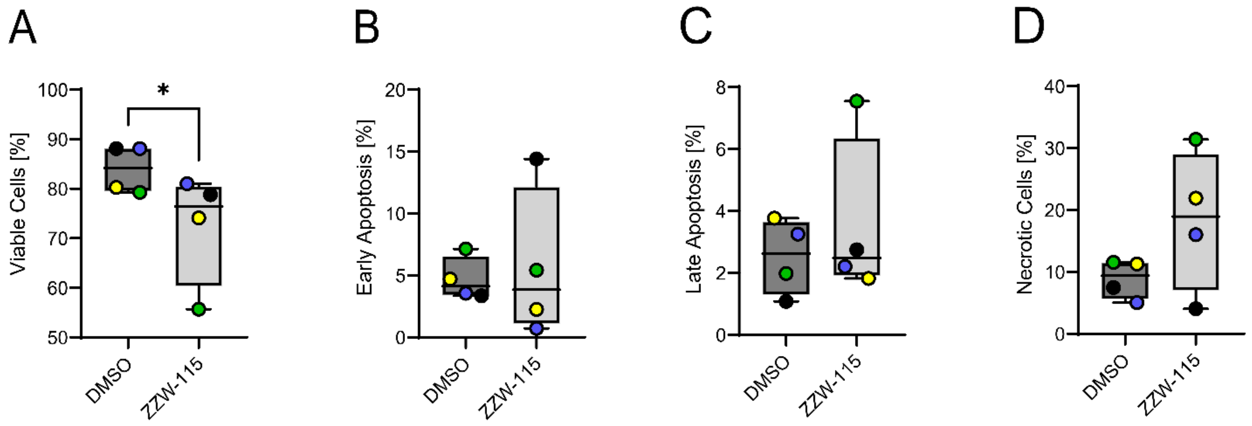

Supplemental Fig. S8: Annexin V/PI analysis and quantification of (A) viable cells (Annexin V-negative/PI-negative), (B) early and (C) late apoptosis as well as (D) necrosis in control phBECs (*n* = 4). Panel A corresponds to Fig. 5L of the main manuscript. The colours represent individual patients. The analysis revealed no significant changes in the levels of early apoptosis, late apoptosis and necrosis between vehicle control (DMSO) and NUPR1-inhibition treatment with 60 µM ZZW-115. However, overall cell viability was significantly decreased (p = 0.0470) due to NUPR1 inhibition. The statistical analysis was performed using repeated measures one-way ANOVA followed by Tukey's post hoc test for multiple comparisons. All data is presented as mean ± SD.

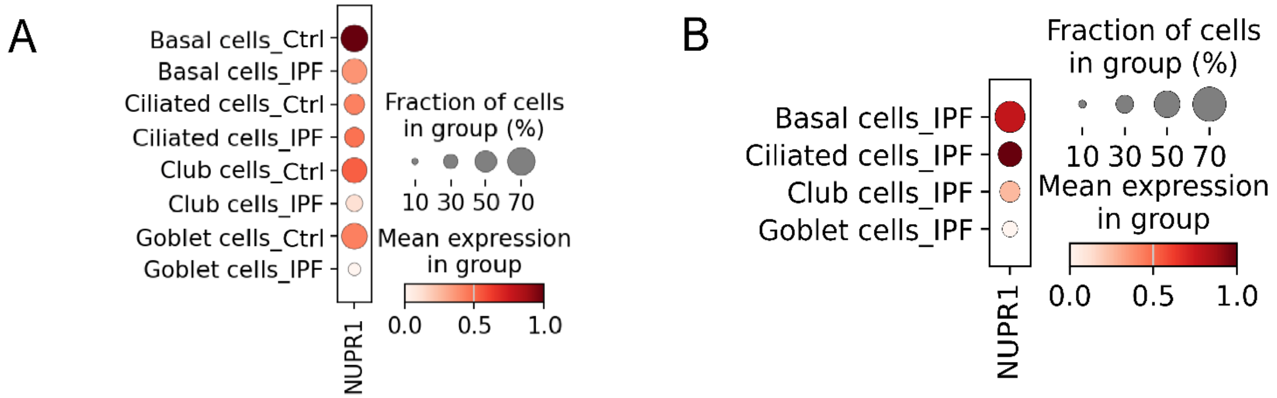

Supplemental Fig. S9: Single cell RNA sequencing of *NUPR1* in bronchial epithelial cells of four combined IPF cohorts. (A) In direct comparison to control bronchial epithelial cells ('Ctrl'), the expression of *NUPR1* in IPF is lower in all cell types, but similar in ciliated cells. **(B)** *NUPR1* expression is highest in ciliated and basal cells, and lowest in secretory cells (club and ciliated cells) in IPF-derived samples.

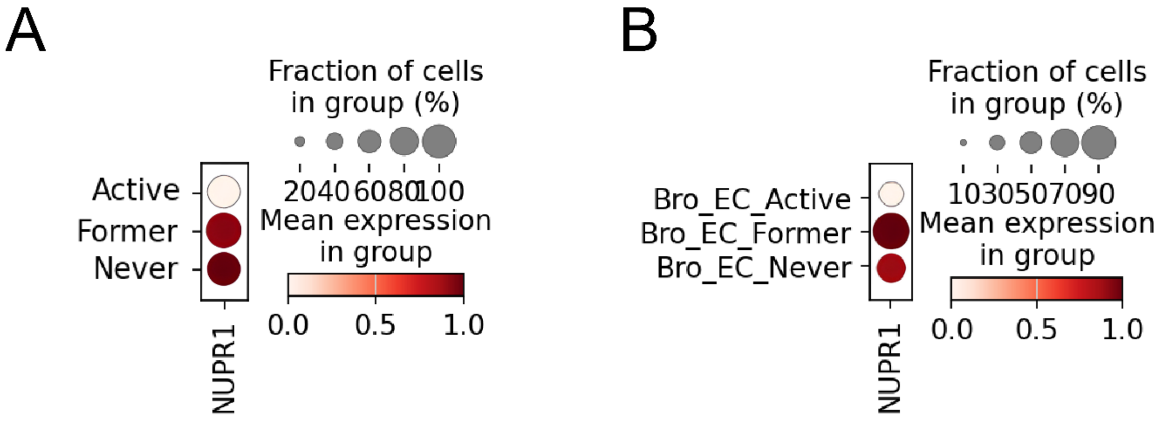

Supplemental Fig. S10: Single cell RNA sequencing (scRNA-seq) of NUPR1 in control samples. (A) ScRNA-seq of four combined cohorts showed that NUPR1 expression is highest in former smokers, followed by never smokers and lowest in active smokers in whole lung and **(B)** bronchial epithelial cells (‘Bro_EC’).

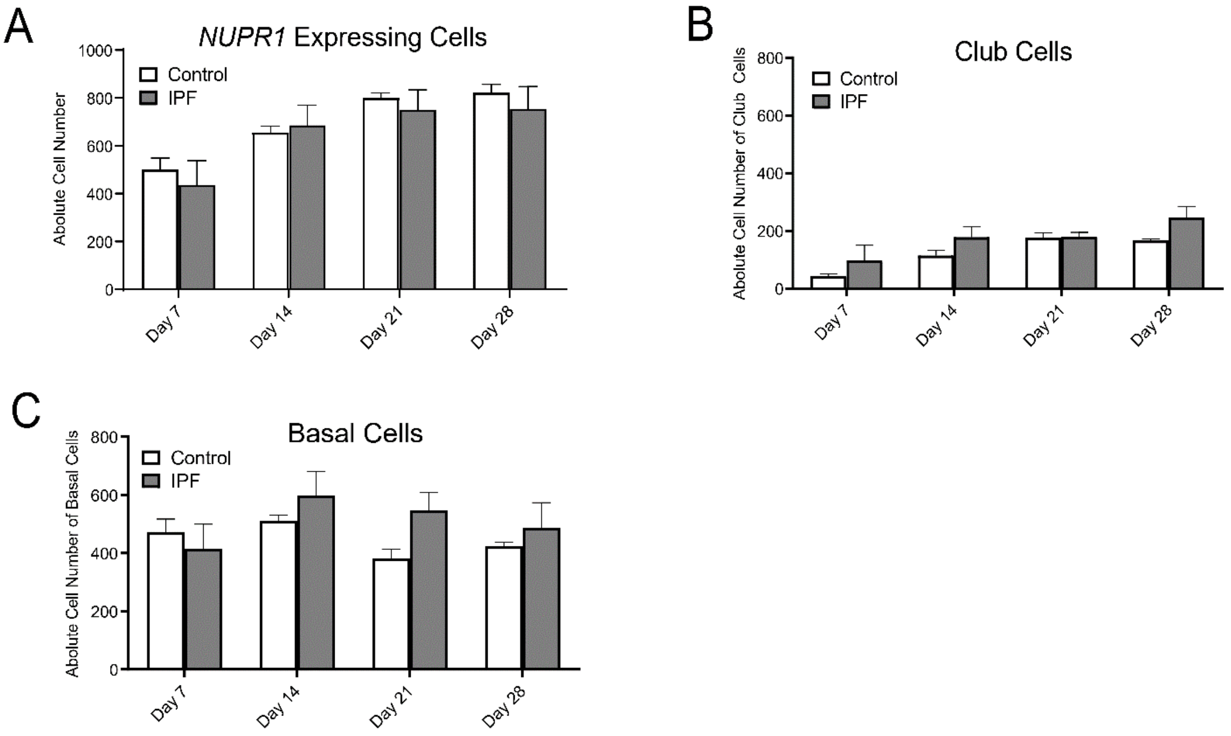

Supplemental Fig. S11: Absolute cell type quantification of control and IPF-derived phBECs at day 7, 14, 21 and 28 (*n* = 4). The quantification of **(A)** NUPR1-positive, which represents a combination of basal and ciliated cells, did not show a significant modulation between control and IPF samples throughout differentiation**. (B)** Club and **(C)** basal cells were not significantly modulated between control and IPF samples. The statistical analysis was conducted using a two-way ANOVA test with multiple comparisons corrected by a two-stage step-up method of Benjamini, Krieger, and Yekutieli. All data is presented as mean ± SD.

4. References

1. Mastalerz, M, et al. Validation of in vitro models for smoke exposure of primary human bronchial epithelial cells. *American journal of physiology. Lung cellular and molecular physiology*1 2022; **322**: L129-L148.
2. Nakayama, M, et al. Quantitative proteomics of differentiated primary bronchial epithelial cells from chronic obstructive pulmonary disease and control identifies potential novel host factors post-influenza A virus infection. *Frontiers in microbiology* 2022; **13**: 957830.
3. Berthold, EJ, et al. Effects of immunophilin inhibitors and non-immunosuppressive analogs on coronavirus replication in human infection models. *Frontiers in cellular and infection microbiology* 2022; **12**: 958634.
4. Schamberger, AC. Staab-Weijnitz, CA. Mise-Racek, N and Eickelberg, O. Cigarette smoke alters primary human bronchial epithelial cell differentiation at the air-liquid interface. *Scientific reports* 2015; **5**: 8163.
5. Mayr, CH, et al. Integrative analysis of cell state changes in lung fibrosis with peripheral protein biomarkers. *EMBO molecular medicine*4 2021; **13**: e12871.
6. Reyfman, PA, et al. Single-Cell Transcriptomic Analysis of Human Lung Provides Insights into the Pathobiology of Pulmonary Fibrosis. *American journal of respiratory and critical care medicine*12 2019; **199**: 1517–36.
7. Habermann, AC, et al. Single-cell RNA sequencing reveals profibrotic roles of distinct epithelial and mesenchymal lineages in pulmonary fibrosis. *Science advances*28 2020; **6**: eaba1972.
8. Adams, TS, et al. Single-cell RNA-seq reveals ectopic and aberrant lung-resident cell populations in idiopathic pulmonary fibrosis. *Science advances*28 2020; **6**: eaba1983.
9. Stoleriu, MG, et al. COPD basal cells are primed towards secretory to multiciliated cell imbalance driving increased resilience to environmental stressors. *Thorax*6 2024; **79**: 524–37.
10. Chakraborty, A, et al. Cholesterol Regulates Airway Epithelial Cell Differentiation by Inhibiting p53 Nuclear Translocation. *International journal of molecular sciences*17 2025; **26**.
11. Perez-Riverol, Y, et al. The PRIDE database at 20 years: 2025 update. *Nucleic acids research*D1 2025; **53**: D543-D553.
12. Johnson, WE. Li, C and Rabinovic, A. Adjusting batch effects in microarray expression data using empirical Bayes methods. *Biostatistics*1 2007; **8**: 118–27.
